# Intermittent glucocorticoid treatment improves muscle metabolism via the PGC1**α**/Lipin1 axis in an aging-related sarcopenia model

**DOI:** 10.1172/JCI177427

**Published:** 2024-05-03

**Authors:** Ashok D. Prabakaran, Kevin McFarland, Karen Miz, Hima Bindu Durumutla, Kevin Piczer, Fadoua El Abdellaoui Soussi, Hannah Latimer, Cole Werbrich, Hyun-Jy Chung, N. Scott Blair, Douglas P. Millay, Andrew J. Morris, Brendan Prideaux, Brian N. Finck, Mattia Quattrocelli

**Affiliations:** 1 Molecular Cardiovascular Biology, Heart Institute, Cincinnati Children’s Hospital Medical Center (CCHMC) and Department Pediatrics, University of Cincinnati College of Medicine, Cincinnati, Ohio, USA.; 2 Department Pharmacology and Toxicology, University of Arkansas for Medical Sciences (UAMS) College of Medicine and Central Arkansas VA Healthcare System, Little Rock, Arkansas, USA.; 3 Department Neuroscience, Cell Biology, and Anatomy, University of Texas Medical Branch (UTMB), Galveston, Texas, USA.; 4 Department of Medicine, Center for Human Nutrition, Washington University in St. Louis, Missouri, USA.

**Keywords:** Aging, Muscle biology, Epigenetics, Mitochondria, Muscle

## Abstract

Sarcopenia burdens the older population through loss of muscle energy and mass, yet treatments to functionally rescue both parameters are lacking. The glucocorticoid prednisone remodels muscle metabolism on the basis of frequency of intake, but its mechanisms in sarcopenia are unknown. We found that once-weekly intermittent prednisone administration rescued muscle quality in aged 24-month-old mice to a level comparable to that seen in young 4-month-old mice. We discovered an age- and sex-independent glucocorticoid receptor transactivation program in muscle encompassing peroxisome proliferator–activated receptor γ coactivator 1 α (PGC1α) and its cofactor Lipin1. Treatment coordinately improved mitochondrial abundance through isoform 1 and muscle mass through isoform 4 of the myocyte-specific PGC1α, which was required for the treatment-driven increase in carbon shuttling from glucose oxidation to amino acid biogenesis. We also probed myocyte-specific Lipin1 as a nonredundant factor coaxing PGC1α upregulation to the stimulation of both oxidative and anabolic effects. Our study unveils an aging-resistant druggable program in myocytes for the coordinated rescue of energy and mass in sarcopenia.

## Abstract 2

Intermittent prednisone coordinates mitochondrial and mass rescue in sarcopenia by transactivating the PGC1α-Lipin1 axis in myocytes.

## Graphical abstract

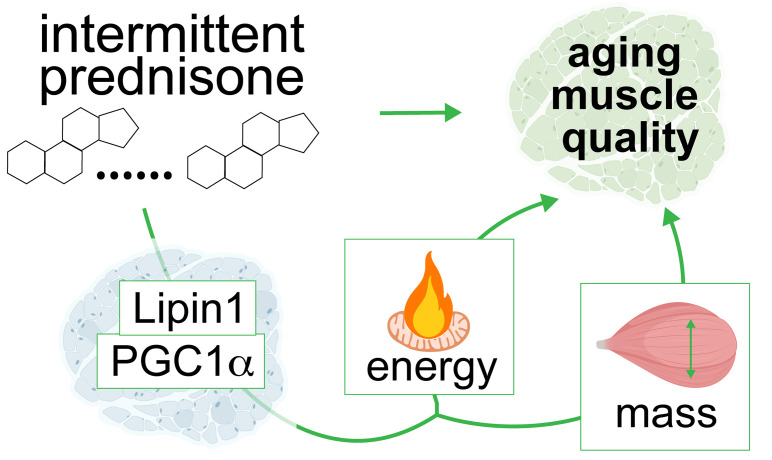

## Introduction

Aging-related sarcopenia contributes to loss of mobility and affects lifestyle in the older population (1). With aging, muscle loses both mass and quality, i.e., its intrinsic capacity to generate force (2). Indeed, sarcopenia correlates with an impaired metabolic capacity to produce energy in muscle (3, 4). However, the reciprocal regulations between metabolic capacity and mass remodeling in muscle aging remain largely unelucidated.

The peroxisome proliferator–activated receptor γ coactivator 1 α (PGC1α) is a major regulator of mitochondrial biology through at least 2 splice variants (5): the canonical longer PGC1α isoform 1 regulates mitochondrial biogenesis and function, whereas the shorter PGC1α isoform 4 (6) increases muscle mass and strength in cachectic muscle (7) and sarcopenia (8). Although the role of PGC1α in mitochondrial capacity (9) and overall mitochondrial protein quality (10) is well established, its effects on age-related sarcopenia and weakness are still debated, and study results are conflicting. Studies in aging transgenic mice reported gain of muscle mass with constitutive PGC1α overexpression (11) and, conversely, loss of lean mass with constitutive muscle PGC1α KO (12). However, another study with constitutive PGC1α overexpression versus PGC1α KO in muscle showed that PGC1α is dispensable for age-related sarcopenia (13). Another recent study showed that lifelong muscle PGC1α overexpression increased muscle mass in male but not female mice and improved muscle fatigue at the expense of specific force (14). Thus, the role of myocyte-specific PGC1α in rescuing age-related sarcopenia and weakness remains unclear. This opens the question of whether additional factors balance the PGC1α action on energy and mass in muscle.

Lipin 1 is a multifunctional protein that regulates muscle function and bioenergetics, and its ablation leads to muscle dysfunction and lipid accumulation in mice (15). Lipin 1 acts in the cytosol as a phosphatidic acid phosphohydrolase (16, 17) and in the nucleus as a regulator of gene transcription (18). In muscle, Lipin1 regulates many complex processes, including myofiber stability and regeneration (19), as well as autophagy and mitophagy (20, 21). In hepatocytes, Lipin1 coactivates PGC1α through a direct protein-protein interaction (18), but this role of Lipin1 remains unexplored in muscle. More generally, the role of the myocyte-specific Lipin1 in muscle aging and energy-mass balance requires further investigation.

Glucocorticoid steroids are potent drugs that regulate both energy metabolism and mass. Dosing frequency of glucocorticoid intake determines the benefit/risk ratio of these drugs with regard to metabolic balance. Chronic, once-daily glucocorticoid intake promotes metabolic imbalance (22). Conversely, dosing intermittence shifts the glucocorticoid metabolic program from pro-wasting, i.e., atrophy and decreased bioenergetics with once-daily prednisone, to pro-ergogenic, i.e., increased bioenergetics and muscle mass with once-weekly prednisone in young adult mice, counteracting the muscle detriments induced by diet-induced obesity (23). In patients with dystrophy, a recent pilot clinical trial reported positive trends in both lean mass and mobility with once-weekly prednisone treatment (24). However, the relevance and myocyte-autonomous mechanisms of glucocorticoid intermittence in the context of muscle aging are still unknown.

Here, we report on the rejuvenating effects of intermittent prednisone on both bioenergetics and mass in the aging muscles of male and female mice. We interrogated transcriptomic and epigenomic data sets to identify activation of the Lipin1/PGC1α axis. We used inducible myocyte-specific KO models for PGC1α and Lipin1 to investigate the requirement of these factors for the coordinated rescue of energy and mass in the absence of developmental or lifelong muscle adaptations to the manipulation of those genes. Moreover, we found that the PGC1α upregulation mediated the boost in amino acid biogenesis from oxidative intermediates, linking the bioenergetic and anabolic stimulations of treatment in muscle. Our study provides evidence and identifies myocyte-specific mechanisms to challenge existing paradigms for glucocorticoid drugs with unexpected antisarcopenic effects.

## Results

### Intermittent, once-weekly prednisone rejuvenates mitochondrial and mass properties of the aging muscle.

**Figure 1:**
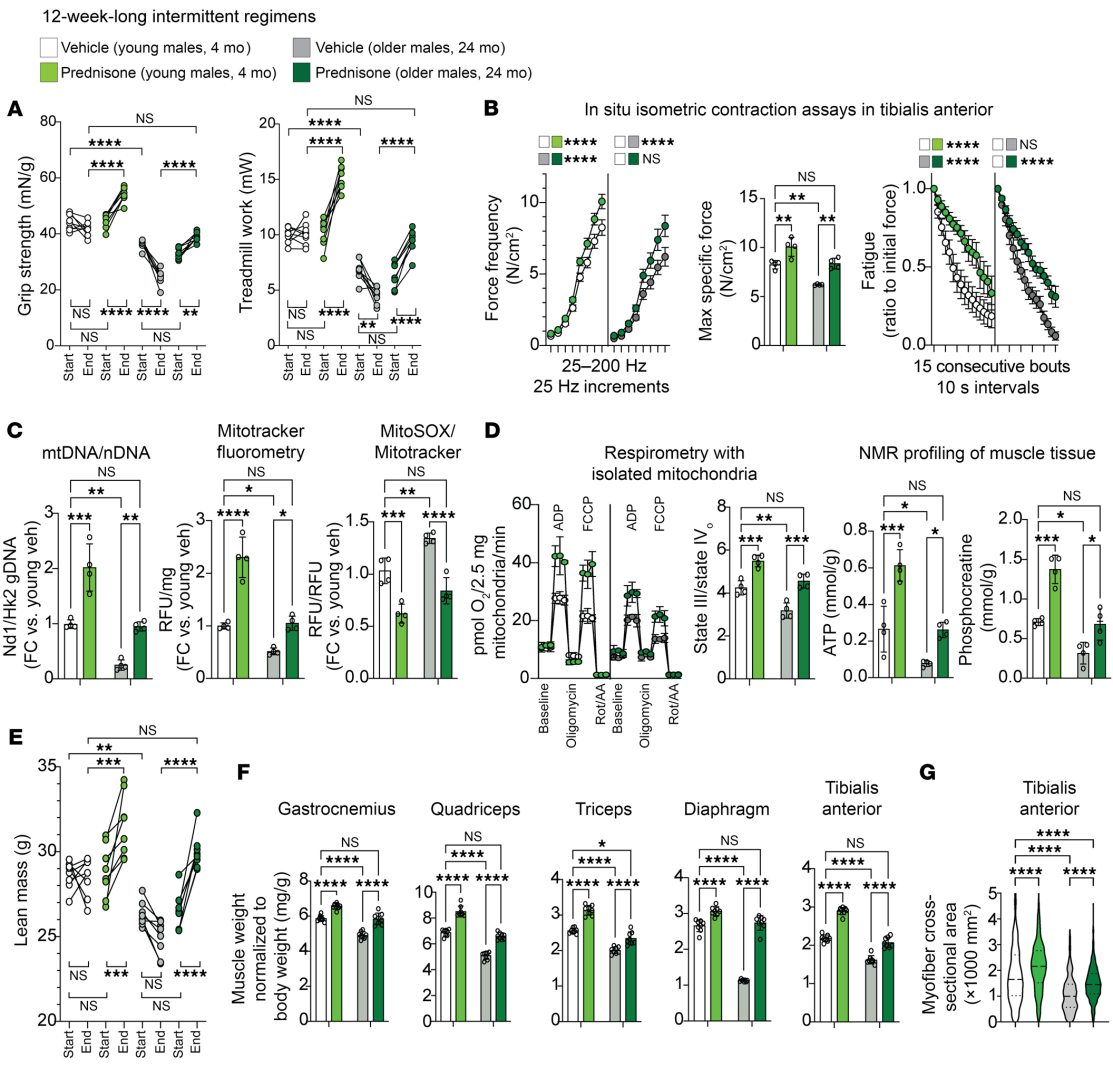
Intermittent once-weekly prednisone regimen rejuvenates mitochondrial and mass properties of aging muscle. (**A**) Treatment improved strength and treadmill performance in background-matched male mice at young adult (4 mo) and older adult (24 mo) ages, improving the parameters of the treated aged mice to levels comparable to those of the control (veh, vehicle) young adult mice at the endpoint. (**B**) Treatment rescued specific force in older mice to levels seen in control young mice, while increasing resistance to repetitive tetanus fatigue to a comparable extent at both ages. max, maximum. (**C** and **D**) Treatment improved mitochondrial abundance (mtDNA/nDNA, MitoTracker) and decreased superoxide levels (MitoSOX) in aged muscle compared with young control-like levels. Analogous trends were observed with mitochondrial respiration levels and NMR-quantitated levels of ATP and phosphocreatine in quadriceps muscles. AA, antimycin A; FC, fold change; rot, rotenone. (**E**–**G**) In treated older mice, total lean mass increased to young control-like levels. This correlated with rescue of muscle weight/body weight ratios in older mice in locomotory (gastrocnemius, quadriceps, triceps) and respiratory (diaphragm) muscles. Tibialis anterior muscle analyses showed coupling of myofiber CSA trends with the changes in muscle mass. *n* = 4–8/group. Histograms and curves report the mean ± SEM; pre- and post-treatment plots report each subject trend; violin plots indicate the mean and the 25th–75th percentiles. *P < 0.05, **P < 0.01, ***P < 0.001, and ****P < 0.0001; (start-end) pre-/post-paired 3-way ANOVA with Šidák’s test (endpoint) (**A** and **E**); 2-way ANOVA with Šidák’s test (**B**–**D**, **F**, and **G**).

Muscle aging is characterized by declines in both mitochondrial capacity and mass (25). On the basis of the initial positive effects we documented on both mitochondrial function and mass in young adult muscle of WT mice (26), we tested the extent to which an intermittent, once-weekly prednisone treatment affected muscle properties in the context of aging. We treated aged WT mice at 24 months of age and background-matched (*C57BL/6JN*) young adult control mice at 4 months of age, all from the National Institute on Aging’s Division of Aging Biology mouse colony. Treatment was consistent with our prior report (26), i.e., once-weekly 1 mg/kg prednisone was given i.p. at zeitgeber time 0 (ZT0) for 12 weeks, controlled by the same schedule of vehicle administration. Pre- and post-treatment noninvasive physiological assessments were conducted 72–24 hours prior to the first drug injection and 24 hours after the last injection, which immediately preceded the invasive assessments. We treated male and female mice in parallel and report the sex-disaggregated data in Figure 1 and Supplemental Figure 1 (supplemental material available online with this article; https://doi.org/10.1172/JCI177427DS1).

As parameters of overall strength and function, we quantitated grip strength and treadmill performance at baseline and after treatment (i.e., ~27 months of age for older mice), and quantitated force production in situ in tibialis anterior muscles after treatment. In the absence of treatment (vehicle), compared with young controls, older mice showed decreased strength and treadmill endurance at the starting point and a decline at the endpoint. Compared with vehicle, treatment increased both parameters in older mice at the endpoint compared with the starting point. The values for treated older mice were not significantly different from the values for the control vehicle-treated young mice at the endpoint. As a validation, the treatment effect was recapitulated in the young mice (Figure 1A). At the endpoint, we used isometric contraction assessments to profile force production through both force frequency and fatigue assays. Treatment improved specific force in older muscle to levels similar to those shown by the control young muscle, while resistance to fatigue was improved by treatment to similar extents in both age groups (Figure 1B).

We then analyzed mitochondrial properties consistently with our previous treatment study (26). We measured relative trends in mitochondrial abundance through mitochondrial DNA/nuclear DNA (mtDNA/nDNA) quantitative PCR (qPCR) quantitation and unbiased MitoTracker fluorometry in parallel in isolated myofibers from flexor digitorum brevis (FDB) muscles. Together with MitoTracker, MitoSOX fluorometry was used to quantitate mitochondrial superoxide production. Treatment increased mitochondrial abundance in both age groups, rescuing the values of the treated older muscles to young control–like levels. Conversely, mitochondrial superoxide production was decreased by treatment in older muscles, suggesting functional coupling in the overall mitochondrial pool after treatment (Figure 1C). This was further elucidated in quadriceps muscle by respirometry curves with isolated mitochondria (fuel: pyruvate) and nuclear magnetic resonance–based (NMR-based) quantitation of the energy exchange molecules ATP and phosphocreatine. Treated aging muscle showed young control–like levels of the ADP-fueled respiratory control ratio (RCR) (state III/state IV_o_) (27) and static levels of ATP and phosphocreatine (Figure 1D).

We then analyzed parameters of lean mass and muscle mass. Using echoMRI, we found that treatment increased overall lean mass in treated older mice to levels similar to those found in young control mice (Figure 1E). The trends in lean mass were matched by analogous trends in muscle/body weight ratios throughout the body, as shown by measurements in 4 different locomotory muscles (gastrocnemius, quadriceps, triceps, and tibialis anterior) and the respiratory muscle diaphragm (Figure 1F). The trends in muscle/body weight ratios were further matched by analogous trends in myofiber cross-sectional area, as shown in the case of tibialis anterior muscle (Figure 1G), further illustrating the treatment-driven rescue of aging muscle mass toward young control–like levels. Discussed so far are the treatment effects in male cohorts, but analogous trends were recorded in parallel for age- and background-matched female cohorts from the same colony (Supplemental Figure 1).

Considering the effects on muscle mass and mitochondrial function, we tested markers of autophagy (LC3B), mitophagy (Pink1), mitochondrial fusion (Mfn2), and fission (Fis1); mitochondrial complex content; independence of muscle mass trends from body weight shifts; and relative abundance and cross-sectional area of myofiber types. Treatment increased LC3B-II/-I and Pink1 protein levels in young and aged muscles, whereas slight changes in Mfn2 and Fis1 were not significant (Supplemental Figure 2A), suggesting that the increases in muscle and mitochondrial abundance were balanced by a compensatory uptick in autophagy and mitophagy without major changes in the mitochondrial fusion/fission cycle. The cumulative mitochondrial complex signal was increased by treatment in whole lysates and mitochondrial fractions of quadriceps muscle samples (Supplemental Figure 2A), consistent with the parallel increases of mitochondrial abundance in whole tissue and respiration capacity in fixed mitochondrial amounts. The trends in overall muscle mass appeared to be independent of body weight shifts, as they were recapitulated when muscle weights were normalized to tibia lengths in a subset of mice (Supplemental Figure 2B). Moreover, we did not record sizable treatment effects in addition to the expected age-related shifts in relative myofiber type abundances in 2 locomotory muscles with mixed fiber typing, i.e., gastrocnemius and triceps, in either males or females (Supplemental Figure 3). In those muscles, aging decreased cross-sectional area (CSA) in type 2B and 2A myofibers, and treatment increased CSA in type 1, 2A, and 2B myofibers (Supplemental Figure 3), consistent with previously reported effects of sarcopenia and exercise-mediated rescue in murine aging myofiber types (28).

Thus, according to the drug schedule and readout parameters tested here, intermittent prednisone treatment “rejuvenated” both mitochondrial function and mass in the aging muscle, i.e., it improved parameters in treated older muscles to young control–like levels.

### Treatment induces a muscle glucocorticoid receptor program that increases PGC1α-Lipin1 expression through aging.

**Figure 2:**
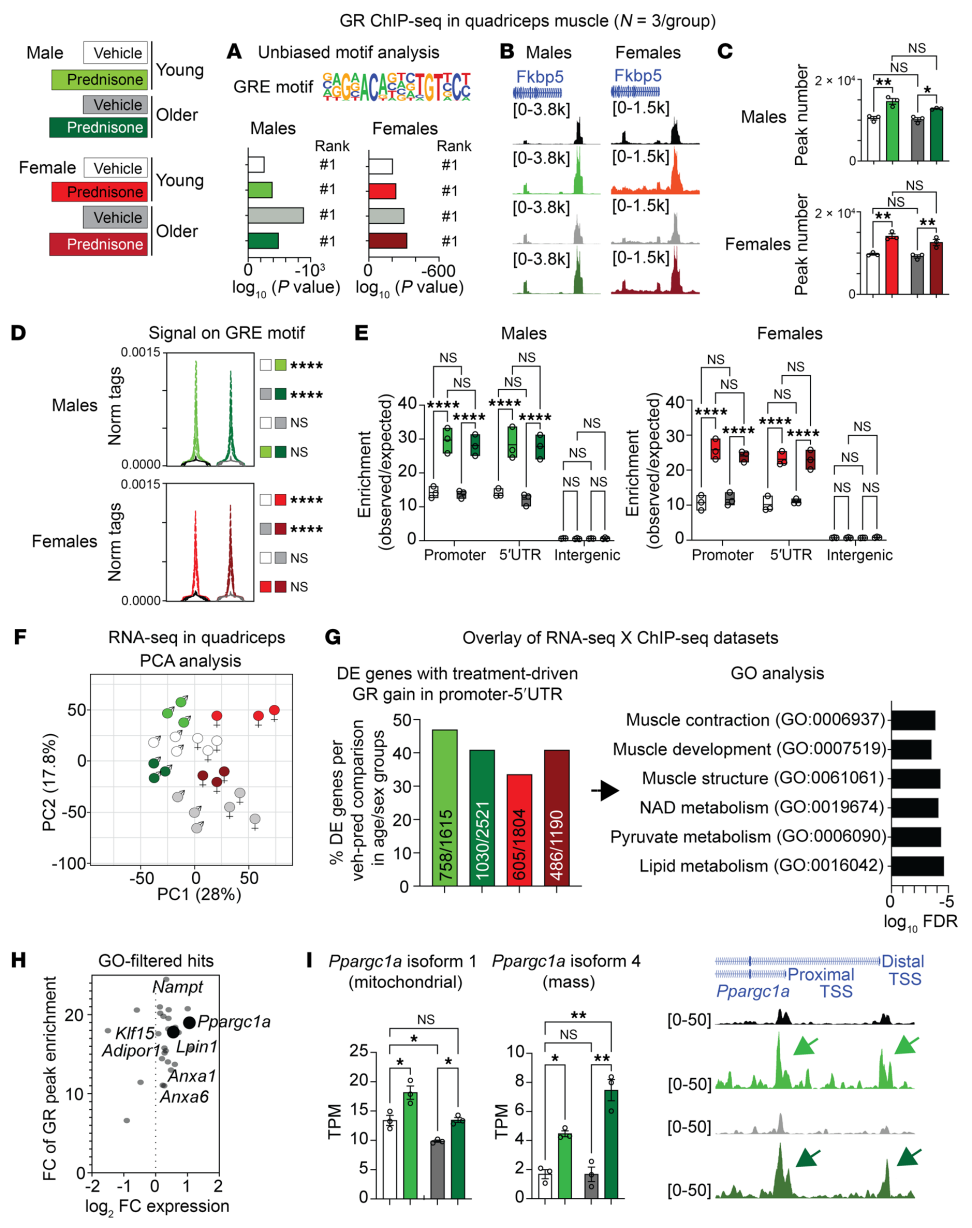
Epigenetic and transcriptional profiling reveals a treatment-induced muscle GR cistrome that is maintained through aging. (**A** and **B**) Motif analysis and robust promoter peaks in the canonical target *Fkbp5* confirm GR ChIP-Seq data sets. (**C**–**E**) Treatment increased GR peak numbers and genome-wide, GRE-bound GR signal to comparable extents in both young and older age groups in both males and females. In all experimental groups, treatment increased the GR signal in promoters and 5′-UTR regions. norm, normalized. (**F**) PCA analysis of RNA-Seq data sets showed age- and treatment-related trends across sexes. (**G** and **H**) GO analysis revealed enrichment for muscle metabolic factors, particularly *Ppargc1a* (encoding PGC1α) and *Lpin1* (encoding the PGC1α cofactor Lipin1). veh, vehicle; pred, prednisone. (**I**) Expression of both isoforms 1 and 4 of *Ppargc1a* was rescued to young-like levels in treated older muscle, correlating with increased GR binding on canonical and alternative start sites (arrows). TPM, transcripts per million. *n* = 3/group. Histograms report the mean ± SEM. *P < 0.05, **P < 0.01, and ****P < 0.0001; 2-way ANOVA with Šidák’s test.

To gain insight into the mechanisms mediating the dual treatment effects on energy and mass in aging muscle, we profiled the epigenomic signal of the glucocorticoid receptor (GR) in all age, sex, and treatment cohorts, in parallel with bulk transcriptomic profiling through RNA-Seq in quadriceps muscles from the same mice. Samples were collected 4 hours after the last drug or vehicle injection. Unbiased motif analysis showed the GR-binding element (GRE) motif to be the top enriched motif in all groups (Figure 2A), and peak tracks showed clear, strong GR peaks upstream of the canonical GR marker *Fkbp5* (Figure 2B), indicating reliable GR ChIP-Seq data sets for further quantitative comparisons.

We first asked whether aging changed the muscle GR epigenomic activity in terms of peak number, signal on GREs, and locus distribution at baseline and after treatment. Treatment increased GR peak numbers and the average GR signal enrichment on the GRE motif genome wide compared with vehicle in both young and aging muscles, but we did not find an age-specific effect in either vehicle- or prednisone-treated muscles (Figure 2, C and D). Similarly, compared with vehicle, treatment increased GR signal in the promoter 5′-UTR rather than in the intergenic regions, but once again we did not find an age-specific effect in this shift (Figure 2E). Also, the trends were comparable in both male and female muscles (Figure 2, C–E).

We next sought to overlay the GR ChIP-Seq data sets with RNA-Seq data sets to identify which GR targets were changing expression levels following treatment in both age groups and both sexes. Principal component analysis (PCA) of the RNA-Seq data sets showed overall sample clustering according to age, treatment, and sex (Figure 2F). We overlaid GR ChIP-Seq and RNA-Seq following this question: How many and which genes are changed by treatment across age and sex groups with regard to differential RNA expression and increased GR signal in their promoter 5′-UTR region?

We found that approximately 40% of the differentially expressed (DE) genes across all age and sex groups had increased GR signal in their promoter 5′-UTR region. When we compiled these gene lists and analyzed them for pathway enrichment through gene ontology (GO), we found several enriched pathways related to muscle regulation and metabolism (Figure 2G). Considering the potential relevance of these pathways to the treatment-induced phenotype across aging, we used these GO pathways, i.e., the genes found to be enriched by GO analysis in these pathways, to filter out potential hits in play here. We confirmed several target genes, including *Anxa1/6* (29), *Klf15* (30), *Nampt* (26), and *AdipoR1* (23) (Supplemental Figure 4A), that we reported to be transactivated by intermittent prednisone treatment in previous studies.

However, we focused on the emergence of *Ppargc1a* (encoding PGC1α) and *Lpin1* (encoding Lipin1) among the top hits (Figure 2H) based on 2 additional findings and literature-informed considerations. On the one hand, running the isoform-specific analyses from our paired-end RNA-Seq data sets, we found that both the canonical mitochondria-regulating isoform 1 and the mass-regulating isoform 4 (7) were increased by treatment in both age groups and, consistent with the idea of double-isoform transactivation, treatment increased GR peaks on both proximal (isoform 1) and distal (isoform 4) *Ppargc1a* transcription start site (TSS) regions (6) in both young and aging muscles (Figure 2I and Supplemental Figure 4B). On the other hand, Lipin1 is a PGC1α cofactor and potentiates PGC1α activity through direct protein-protein interaction (18). We found that treatment increased GR transactivation of *Lpin1* in mice of both ages, rescued the aging-induced decrease in *Lpin1* expression in muscle (Supplemental Figure 5A), and rescued the levels of PGC1α binding to Lipin1 (Supplemental Figure 5B). Moreover, in each of the sequences underlying the identified GR peaks, we found a canonical GRE (ACAnnnTGT). Through luciferase assays with control and GRE-deleted constructs, we found that all 3 ChIP-Seq identified GR-bound GREs were responsive to prednisone in vitro in C2C12 myoblasts through the GRE sequence (Supplemental Figure 5C). Furthermore, the oxidative boost dependent on the Lipin1-PGC1α interaction correlates with a decrease in triacylglycerol (18). We confirmed this in a subset of control versus treated quadriceps muscles in the aged male cohort through untargeted lipidomics, which revealed a remarkable decrease across 47 triacylglycerol species in treated aged muscles (Supplemental Figure 6A).

Thus, epigenomic and transcriptomic profiling identified a GR program that is elicited by intermittent prednisone and regulates muscle function and metabolism across aging. Intriguingly, we found marked GR transactivation of PGC1α-Lipin1, and, in the next experiments, we sought to determine their role in the rescue of aging muscle enabled by treatment.

### Muscle PGC1α is required by intermittent prednisone to coordinately stimulate energy and mass in muscle.

To probe the extent to which PGC1α mediates the effects of chronic intermittent prednisone in muscle, we generated mice with myocyte-specific inducible deletion of PGC1α by crossing *Ppargc1a^fl/fl^* mice (31) with *ACTA1-MerCreMer^+^* mice (32) on the C57BL/6J background. This background is slightly different than that of the WT mice used in the young/aged cohorts, but (a) it was consistent with all our other transgenic lines, including the Lipin1 KO also used in this study, and (b) the nonablated control mice on this background recapitulated all the treatment features that we described above for mice on the 6JN background (see below). PGC1α ablation was induced in mice starting at 3 months of age using i.p. (20 mg/kg per day for 5 days) and then chow-mediated intake (40 mg/kg) of tamoxifen for 14 days, followed by 14 days of washout. These conditions allowed us to reduce PGC1α levels in whole quadriceps muscle lysates by approximately 85%, as we reported before (26). In this study, we compared *Cre^+/–^* *Ppargc1a^WT/WT^* (PGC1α WT) mice with *Cre^+/–^* *Pparcg1a^fl/fl^* (PGC1α-KO) male littermates after tamoxifen/washout to take into account both tamoxifen and Cre presence in both cohorts. After ablation/washout, the mice were started on 12-week-long regimens of intermittent prednisone or vehicle treatment from 4 months of age, the same age and treatment conditions for the young cohorts of the previous experiment. We used this timeline to minimize the adult muscle adaptations to gene ablation before treatment effects. Given the epigenomic and transcriptomic screening of the initial cohorts with targets common to both sexes, in these subsequent mechanistic experiments, we focused on only 1 sex (males) to maintain power to detect trends while decreasing the overall number of mice.

**Figure 3:**
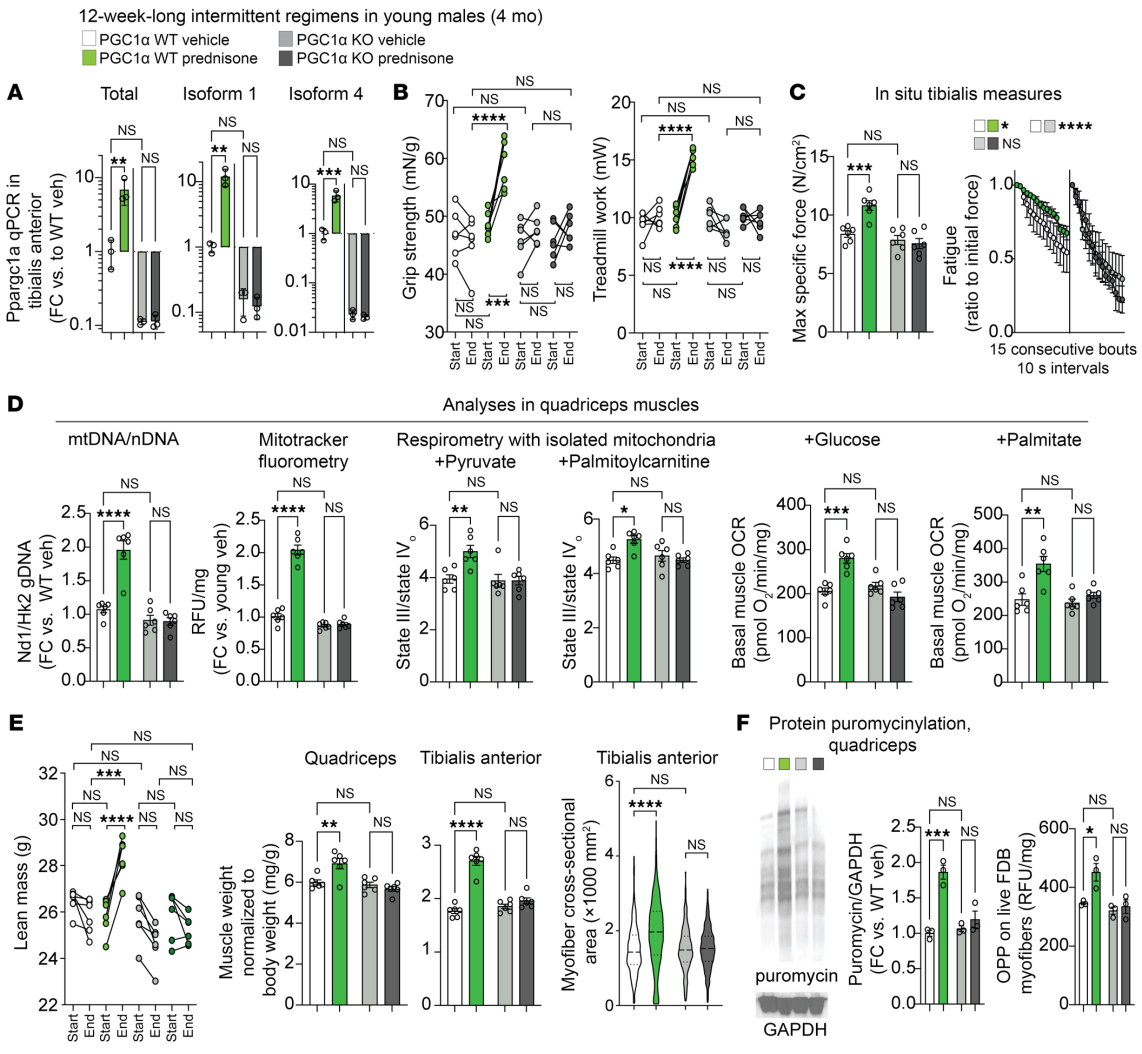
Myocyte-specific inducible PGC1α ablation blocks treatment effects on both mitochondrial function and muscle mass. (**A**) Recombination of the floxed allele reduced expression of both PGC1α isoforms in muscle. (**B** and **C**) In young adult mice, myocyte-specific inducible PGC1α ablation blocked the effects of 12-week-long intermittent prednisone treatment on strength, treadmill performance, force, and fatigue. (**D**) PGC1α ablation blocked or blunted the treatment effects on mitochondrial abundance and on the mitochondrial RCR and the basal OCR in muscle tissue regardless of fuel. (**E**) PGC1α ablation blocked or blunted treatment effects on lean mass, muscle mass, myofiber CSA. (**F**) Treatment increased protein translation in muscle dependent on myocyte PGC1α. *n* = 3–6/group. Histograms and curves report the mean ± SEM; pre- and post-treatment plots report each subject trend; violin plots indicate the mean and 25th–75th percentiles. *P < 0.05, **P < 0.01, ***P < 0.001, and ****P < 0.0001; (start-end) pre-/post-paired 3-way ANOVA with Šidák’s test (**B** and **E**); (endpoint) 2-way ANOVA with Šidák’s test (**A**, **C**, **D**, and **F**).

The *Pparcg1a*-floxed allele features *loxP* sites surrounding exons 3–5, which are shared by the transcripts of both isoforms 1 and 4 (7). We therefore verified that both transcripts were deleted in the PGC1α-KO muscles at baseline. Compared with WT PGC1α, PGC1α-KO muscles showed profound downregulation of total *Ppargc1a* expression, as well as of isoform 1 and isoform 4 transcripts (Figure 3A), as assessed through qPCR using previously reported discriminating primers (7). Regarding overall function, despite a lack of differences induced by genotype at baseline and the endpoint (i.e., WT vehicle vs. KO vehicle), PGC1α ablation blocked the treatment effect in both grip strength and treadmill tests (Figure 3B). With regard to force production, PGC1α ablation did not change specific force but did decrease fatigue resistance in vehicle-treated mice and further blocked the treatment effect on increases in both parameters (Figure 3C).

Previously, we found that myocyte PGC1α is required for the mitochondrial effects of a single circadian-specific glucocorticoid pulse (26). We therefore checked whether this was still true for the chronic intermittent prednisone treatment effects on muscle mitochondria. Indeed, consistent with the finding of PGC1α transactivation by chronic treatment, we found that PGC1α ablation blocked the treatment effect on mitochondrial abundance, the RCR of isolated mitochondria, and the basal oxygen consumption rate (OCR) of quadriceps muscle tissue (Figure 3D). The KO effects on respiration appeared to be related to overall mitochondrial function rather than shifts in nutrient preference, as they were recapitulated with either glucose/pyruvate or palmitate/palmitoylcarnitine as fuels (Figure 3D; see also respirometry curves in Supplemental Figure 6B).

Strikingly, PGC1α ablation also blocked the treatment effects on lean and muscle mass. Despite no genotype differences in vehicle-treated mice, PGC1α ablation blocked the treatment effect on lean mass, muscle/body weight ratio (shown here for both muscles used for analyses in this model), and myofiber cross-sectional area (Figure 3E). Moreover, the treatment/KO effects appeared to be related to anabolic propensity, as shown by 2 independent puromycin-based assays of protein synthesis: protein puromycinylation in quadriceps muscle lysates after in vivo puromycin injection and ex vivo *O*-propargyl-puromycin incorporation/fluorometry (33) in live myofibers (Figure 3F). We detected no significant effects of muscle PGC1α ablation at the starting point or endpoint, or of treatment on fat mass, body weight, or food and water intake (Supplemental Figure 6C).

Thus, inducible PGC1α ablation in adult myocytes without long-term and/or developmental adaptations blocked the effects of chronic intermittent prednisone treatment, not only on mitochondrial capacity, but on muscle mass as well.

### PGC1α mediates the treatment-induced increase in carbon shuttling between oxidative intermediates and amino acids in muscle.

We were intrigued by the fact that the myocyte-specific PGC1α mediated both mitochondrial and mass effects of treatment. We therefore hypothesized that the upregulated PGC1α mediated a myocyte-autonomous metabolic program coordinating the increased mitochondrial metabolism with anabolic growth. Amino acid availability determines mass rescue in sarcopenia (34). Intermediary metabolites like pyruvate and TCA cycle intermediates are direct precursors of amino acids like alanine, glutamine, and aspartate, which are generally decreased in aging muscle (35). We therefore asked whether PGC1α coordinates the carbon shuttling from glucose to amino acid biogenesis in treated muscle. To investigate this in our transgenic muscles, we traced the glucose contribution to in-muscle amino acids using an ex vivo system in which the isolated muscle undergoes repetitive contractions in the presence of ^13^C-labeled glucose and insulin (30). Albeit not in steady state, this system offers the advantage of quantitating muscle-autonomous effects without circulating and extra-muscle contributions, and we previously used it to trace macronutrient fate, including that of glucose, after intermittent prednisone treatments in dystrophic muscles (30) and normal versus obese WT muscles (23).

**Figure 4:**
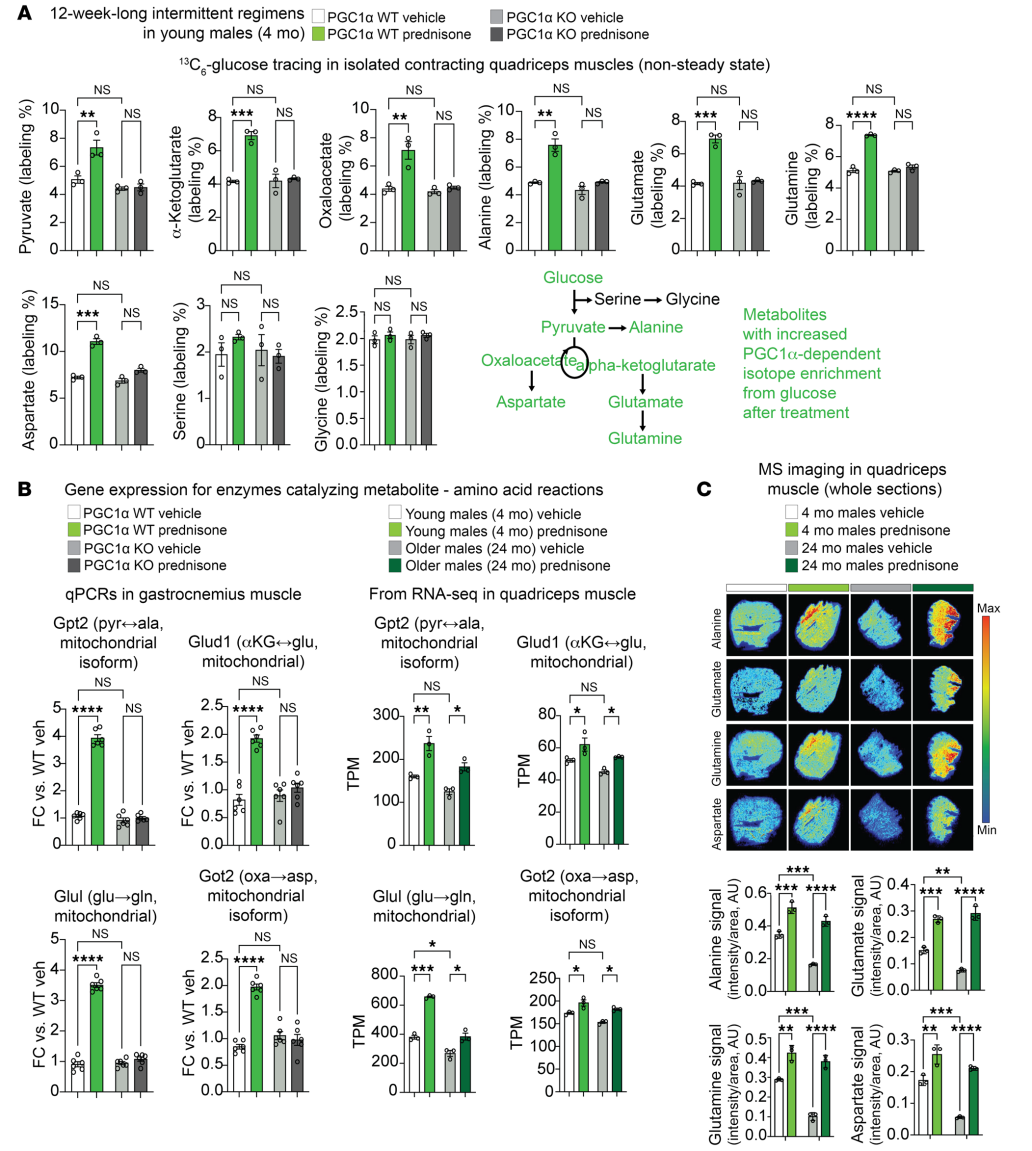
Treatment increases carbon shuttling between glucose and amino acids in muscle dependent on myocyte-specific PGC1α. (**A**) In isolated contracting muscle exposed to ^13^C_6_-glucose, treatment increased carbon shuttling to alanine, glutamate, glutamine, and aspartate, but not to serine or glycine. (**B**) Myocyte-specific PGC1α was required for treatment-driven upregulation of mitochondrial enzymes and/or enzyme isoforms mediating the underlying reactions between glucose derivatives and amino acids. The treatment effect on expression of those genes was also confirmed in young and older muscles by RNA-Seq. αKG, α-ketoglutarate; ala, alanine; asp, aspartate; glu, glutamate; gln, glutamine; oxa, oxaloacetate; pyr, pyruvate. (**C**) MS images showed increased levels of target amino acids in treated young and aged muscles after a glucose-plus-insulin challenge. *n* = 3–6/group. Histograms report the mean ± SEM. *P < 0.05, **P < 0.01, ***P < 0.001, and ****P < 0.0001; 2-way ANOVA with Šidák’s test.

We compared WT PGC1α and PGC1α-KO muscles after vehicle or prednisone treatment for labeling rates of glucose intermediates (pyruvate, α-ketoglutarate, oxaloacetate) and their putative amino acid products (alanine, glutamate/glutamine, aspartate) downstream of uniformly labeled ^13^C_6_-glucose. We also quantitated labeling rates for amino acids produced from glycolytic intermediates (serine, glycine). For each metabolite, we quantitated overall fractional labeling, i.e., the percentage of the sum of all ^13^C-labeled isoforms versus the total sum of labeled and unlabeled isoforms. Compared with vehicle controls, treated WT PGC1α muscles showed increased labeling of the glucose intermediates and their amino acid products, but the treatment effect was blocked or blunted with myocyte PGC1α ablation (Figure 4A). In accordance with the effects of muscle PGC1α overexpression or KO on lactate dehydrogenase isoforms (36), in WT PGC1α muscle treatment (PGC1α upregulation) decreased labeling and lactate levels, whereas both parameters were increased over WT control levels in both treated and untreated PGC1α-KO muscle (Supplemental Figure 6D). No significant treatment or genotype effects were quantitated for labeled serine and glycine (Figure 4A), and PGC1α ablation did not significantly affect the treatment-driven increase in overall glucose tolerance (Supplemental Figure 6E), supporting the notion of a specific pathway rather than a boost in overall glucose use.

In light of a prior report implicating PGC1α in the transcriptional control of the mitochondrial alanine transaminase (37) (gene name, *Gpt2*), we asked whether we could quantitate a PGC1α-dependent effect on the mitochondrial enzymes or mitochondrial enzyme isoforms mediating the carbon shuttling between the metabolites and amino acids found enriched in labeling. Therefore, in addition to *Gpt2* (pyruvate ↔ alanine), we quantitated the expression levels of *Glud1* (α-ketoglutarate ↔ glutamate), *Glul* (glutamate → glutamine), and *Got2* (oxaloacetate → aspartate) in the gastrocnemius muscles of the same animals whose quadriceps muscles were used for the glucose-tracing experiments. For all 4 of those enzymes, PGC1α ablation blocked or blunted the treatment-driven upregulation seen in WT PGC1α muscle (Figure 4B, left; Supplemental Figure 6F). Accordingly, we checked against our RNA-Seq data sets in young and older mice and found that the same 4 enzyme genes were upregulated by treatment in muscles of mice in both the young and older age groups (Figure 4B, right). We tested whether the treatment effect on glucose-derived amino acid biogenesis was quantifiable in young and aged muscle. Twenty-four hours after the last treatment injection, we challenged the young and aged mice with 1 g/kg glucose coupled with 0.5 U/kg insulin to maximize muscle glucose uptake across ages and treatment groups. MS imaging of cryosections from quadriceps muscles collected 2 hours after challenge showed increased levels of alanine, glutamate, glutamine, and aspartate in both young and aged muscles (Figure 4C).

Thus, myocyte-specific PGC1α mediated the metabolic program enabled by intermittent prednisone treatment in muscle to coordinate mitochondrial metabolism with amino acid biogenesis.

### Myocyte-specific Lipin1 is required for the proergogenic effects of treatment upstream of PGC1α.

**Figure 5:**
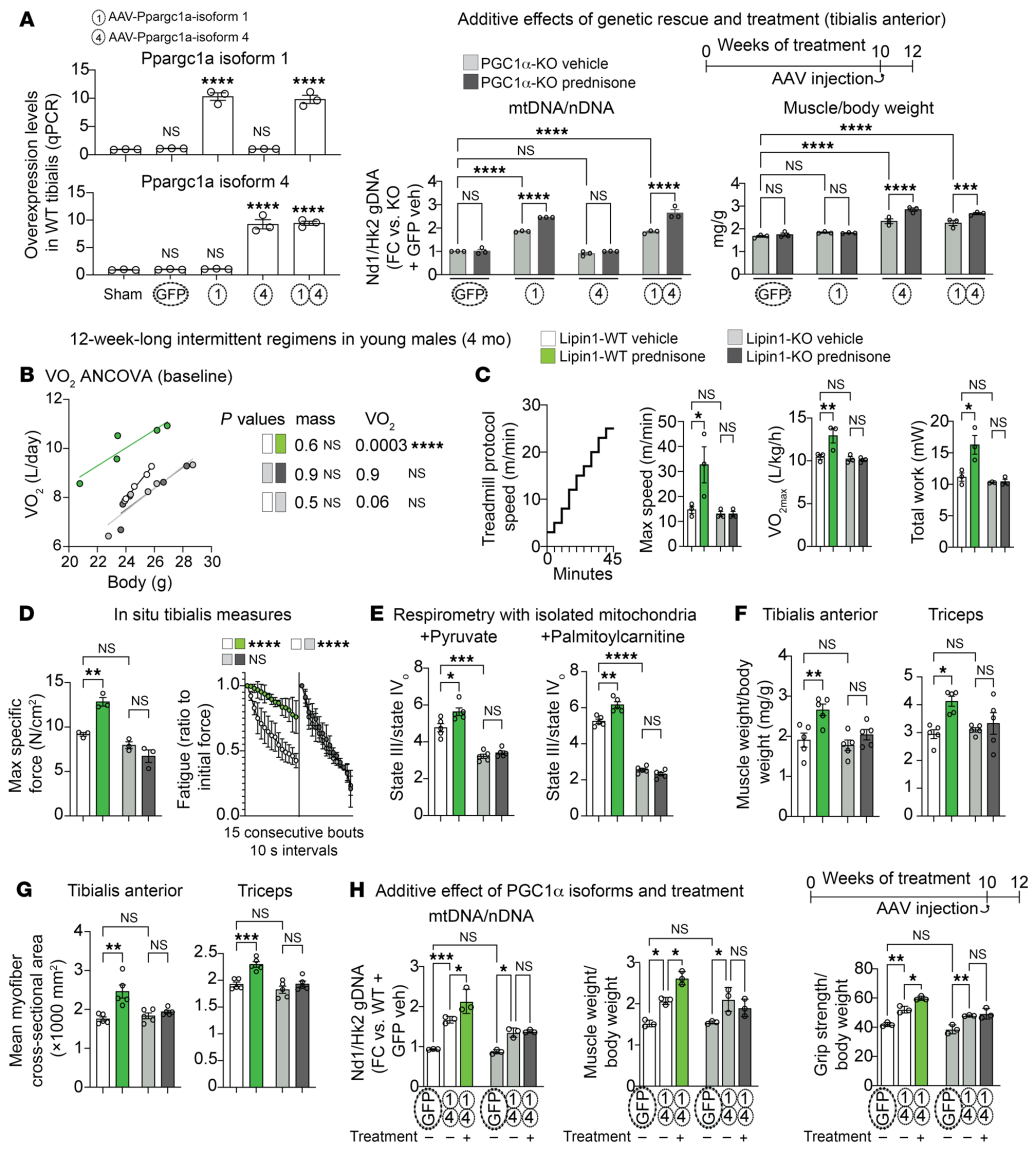
Myocyte-specific Lipin1 controls energy-mass balance in muscle. (**A**) MyoAAV-mediated overexpression in WT muscle 2 weeks after r.o. injection of 10^12^ vg/mouse (left). Combination of AAV and treatment in PGC1α-KO mice revealed an additive effect of the treatment on genetic rescue of mitochondrial abundance by Pgc1α isoform 1 and muscle mass by PGC1α isoform 4 (right) in tibialis anterior muscles. Together with our RNA-Seq/ChIP-Seq screening, the additive effect warranted investigation of Lipin1 as a treatment-driven cofactor to coax PGC1α regulation with energy-mass balance. (**B**) ANCOVA for VO_2_ in metabolic cages without specific exercise triggers showed increased VO_2_ independent from body mass in control (WT Lipin1) mice, but not after Lipin1 ablation (Lipin1-KO). (**C**) In the metabolic treadmill test, treatment increased VO_2max_ as well as speed and work until exhaustion dependent on myocyte-specific Lipin1. (**D** and **E**) Lipin 1 was critical for treatment-driven effects on muscle force and fatigability and mitochondrial respiration. (**F** and **G**) Analogously to its cofactor PGC1α manipulation, Lipin1 ablation blunted or blocked treatment effects on muscle mass in 2 different locomotory muscles (tibialis, hind limbs; triceps, forelimbs). (**H**) KO of Lipin1 blocked the additive effect of treatment on top of the PGC1α isoform 1 and isoform 4 overexpression effect on mitochondrial abundance, tibialis anterior muscle mass, and grip strength. *n* = 3–5/group. Histograms and curves report the mean ± SEM. *P < 0.05, **P < 0.01, ***P < 0.001, and ****P < 0.0001; 2-way ANOVA with Šidák’s test.

Considering the combined upregulation of *Ppargc1a* isoforms 1 and 4 following treatment, we asked whether each isoform was sufficient to rescue a specific parameter of treatment effect, i.e., mitochondrial abundance by isoform 1 and muscle mass by isoform 4. We generated adeno-associated viruses (AAVs) to overexpress either GFP (control), *Ppargc1a* isoform 1, or isoform 4 downstream of a *CMV* promoter. Adult myocyte tropism was promoted by using the MyoAAV serotype (38) and confirmed via qPCR in WT muscle tissue 2 weeks after a single retro-orbital (r.o.) injection of 10^12^ vg/mouse (Figure 5A, left). We then used PGC1α-KO mice for a genetic rescue experiment with AAV overexpression of isoform 1, isoform 4, or both in vehicle versus treatment conditions. On the one hand, isoform 1 was sufficient to enable the treatment effect on mitochondrial abundance but not mass, consistent with the effect of this isoform on oxidative efficiency (39). On the other hand, isoform 4 was sufficient to enable the treatment effect on muscle mass but not mitochondrial abundance, consistent with prior reports (7). Importantly, the treatment showed a significant additive effect over the genetic rescue effect (Figure 5A, right). Through cryohistology in tibialis anterior muscles and hydroxyproline dosing in gastrocnemius muscles, we detected no sizable changes in muscle architecture or fibrosis of the AAV-transduced cohorts (Supplemental Figure 7A). In addition to our inducible KO data, this rescue experiment confirmed the specific roles of PGC1α isoforms 1 and 4 in the combined energy-mass effect of intermittent prednisone treatment. However, the additive effect of treatment over AAV reexpression led us to hypothesize that an additional factor induced by treatment is required to fully coax PGC1α to achieve the global antisarcopenic effect.

Within that hypothesis, we were intrigued by the emergence of Lipin1 as a GR transactivation target with intermittent prednisone in tandem with PGC1α in aging muscle. Indeed, prior experiments in liver showed that Lipin1 is a direct coactivator of PGC1α through protein-protein interaction and primes it to enhance its prometabolic gene program (18). Constitutive KO of Lipin1 impairs muscle function and mitochondrial metabolism in young and mature adult ages (15). In the context of intermittent prednisone effects, we reasoned that if PGC1α mediates energy-mass coordination and Lipin1 is a critical PGC1α coactivator, then Lipin1 could also be required for treatment effects.

We generated transgenic mice for myocyte-restricted inducible ablation of Lipin1 by crossing *Lpin1^fl/fl^* (floxed exon7, complete protein loss) mice (15) with *ACTA1-MerCreMer^+^* mice (32) on the C57BL/6J background. We used the same tamoxifen/washout protocol as for PGC1α-KO and found approximately 85% Lipin1 ablation in quadriceps muscle (Supplemental Figure 7B). Consistent with the PGC1α-KO experiments, we compared *Cre^+/–^* *Lpin1^WT/WT^* (WT Lipin1) versus *Cre^+/–^* *Lpin1^fl/fl^* (Lipin1-KO) male littermates for the effects of a 12-week regimen of intermittent prednisone or vehicle treatment from 4 months of age.

Considering the potential involvement of Lipin1 in muscle oxidative capacity, we interrogated the extent to which the myocyte-specific Lipin1 regulates body-wide VO_2_ at rest and during aerobic exercise of mice in metabolic cages and on the treadmill. Indeed, aging-related exercise intolerance is evident in mice through decreases in baseline and maximal VO_2_ values (40). At rest, intermittent prednisone increased VO_2_ independent of body mass, whereas inducible Lipin1 ablation induced a downward trend in the vehicle-treated muscles and further blocked the treatment effect (Figure 5B). We then tested untrained mice through acute exercise with a stepwise speed ramp-up on a metabolic treadmill. We found that the treatment significantly increased the maximal values of speed, VO_2_, and work before exhaustion in WT Lipin1 mice but not Lipin1-KO mice, suggesting a treatment-driven increase in aerobic fitness dependent on muscle Lipin1 (Figure 5C).

In parallel to VO_2_ and exercise capacity trends, Lipin1 ablation blocked the treatment effect on specific force and fatigue, decreasing the resistance to fatigue also in vehicle-treated muscle (Figure 5D). Furthermore, Lipin1 deletion blocked the treatment effect on ATP and phosphocreatine levels (Supplemental Figure 7C) as well as on the mitochondrial RCR (Figure 5E). Consistently with the KO-driven effects on mitochondrial respiration, we found increased *Pdk4* and decreased *Cpt1b* expression in Lipin1-KO versus WT muscles (Supplemental Figure 7D). Intriguingly and in line with our PGC1α findings and Lipin1-PGC1α protein-protein interaction findings, Lipin1 ablation also blocked the treatment effect on the muscle mass parameters of muscle/body weight ratios and myofiber cross-sectional area in 2 different locomotory muscles (tibialis, hind limbs; triceps, forelimbs; Figure 5, F and G) without sizable shifts in myofiber typing (Supplemental Figure 7E). These effects were achieved even though Lipin1 ablation did not significantly change the treatment effect on the upregulation of muscle PGC1α isoforms compared with vehicle control (Supplemental Figure 7F).

To revisit our MyoAAV-based experiment with PGC1α isoforms, we used Lipin1-KO mice to test the hypothesis that Lipin1 is indeed the nonredundant additional factor underlying the additive treatment effect over PGC1α isoform upregulation. We repeated the combined overexpression of isoforms 1 and 4, with and without treatment, in Lipin1-KO versus WT mice using the same timeline and conditions as in the prior experiment in PGC1α-KO mice. We monitored mtDNA/nDNA and muscle weight/body weight ratios as indicators of mitochondrial abundance and muscle mass, and grip strength/body weight ratios as an indicator of muscle strength. In the presence of MyoAAV-driven upregulation of the PGC1α isoforms, we found that treatment induced an additive effect in all 3 of these parameters compared with vehicle only in WT Lipin1 but not Lipin1-KO mice (Figure 5H), confirming the nonredundant role of Lipin1 in the treatment effect.

Thus, Lipin1 is a GR transactivation target in aging muscle with intermittent prednisone treatment and a key factor in the treatment-PGC1α muscle program.

## Discussion

In aggregate, our data show that exogenous glucocorticoid intermittence can rescue both mitochondrial and mass defects in the aging muscle through the PGC1α/Lipin1 axis. Based on the functional improvements recapitulated by treatment to a variable extent in aging mice of both sexes, we focused here on “inclusive” gene programs underlying comparable remodeling across ages and sexes. However, because sex-specific differences can be identified in specific molecular markers of intermittent prednisone effects in young adult mice (41), future studies are warranted to further investigate how aging affects the “exclusive,” sex-dimorphic programs enabled by glucocorticoid intake.

Our findings linking the pro-ergogenic GR program to simultaneous upregulation of both “mitochondrial” and “mass” *Pparcg1a* isoforms argue in favor of 2 additional concepts for the puzzling role of PGC1α in muscle aging. First, our data demonstrate that a balanced upregulation of both isoforms promotes the balanced rescue of both mitochondrial capacity and muscle mass in the context of sarcopenia. Second, the GR engagement on both isoform TSSs by the glucocorticoid regimen we used here (1 mg/kg prednisone once weekly at ZT0) implicates the myocyte GR as a context-specific additional factor that coaxes the PGC1α isoform regulation with muscle remodeling outputs. Regarding both aspects, our data raise several compelling questions for the aging muscle, including shared/differential mechanisms of PGC1α isoforms and which cofactors are engaged by the GR in beneficial versus deleterious muscle contexts. Our findings are consistent with genetic experiments with PGC1α isoforms (6–8, 39). The role of PGC1α in mitochondrial proteostasis has emerged as an important determinant of muscle health and exercise efficacy (10). Future studies are warranted to pinpoint the effects of prednisone intermittence on proteostasis in sarcopenia (42).

Furthermore, our study identifies an additional, nonredundant factor required for coaxing PGC1α activation toward energy-mass rescue in sarcopenia, i.e., Lipin1. Our data implicate *Lpin1* as a GR transactivation target in muscle and required nonredundant factor to link PGC1α to the pro-ergogenic program enabled by glucocorticoid intermittence in the aging muscle. Considering the recent findings with Lipin1 ablation and complex lipid metabolism in heart (43), future studies are warranted to better identify how Lipin1 regulates lipotoxicity in sarcopenia.

Glucose metabolism contributes to cell mass during development (44) and — as highlighted by our data — also in aging. The role of the mitochondrial TCA cycle in providing the building blocks for many anabolic pathways is well known (45), yet its role in aging muscle still needs further elucidation. An important question to address in the future will be how glucocorticoid intermittence regulates the relationship between nonessential and essential amino acid availability, an important point for mass regulation and exercise tolerance in the aging muscle (46).

### Limitations of the study.

Our study has important limitations to keep in mind when interpreting our findings, especially when projecting their translational potential. We used aging WT mice between 24 and 27 months of age as the main model of sarcopenia. While they recapitulate several key hallmarks of aging (47), aging mice do not fully mimic the extraordinarily complex biology of human aging. Our omics analyses were limited by the small sample number of 3 per group. However, we note that the overlap between transcriptomic and epigenomic data sets empowered our data sets to identify genetically actionable, myocyte-autonomous targets thanks to the convergence-based analysis, i.e., filtering for convergent treatment trends in young and old male and female mice. We recognize that 3 samples per group is insufficient to identify sex-specific differences, which indeed we did not address here. We also acknowledge that our isolated muscle force analyses did not focus on calcium handling, which remains an important parameter to directly address in future studies with glucocorticoids in muscle aging. Also, an important consideration for our mechanistic studies with PGC1α and Lipin1 is that our experiments with myocyte-specific inducible ablation were conducted in 4-month-old mice (young adulthood). This is because our most promising functional treatment phenotypes and epigenomic-transcriptomic screening hits in the aging cohorts were recapitulated in the young adult cohorts too. However, we recognize that this is most likely an oversimplification and that age-matched ablation studies, i.e., ablation at 24 months of age, will shed light on additional molecular mechanisms at play here.

In summary, our study reports on the thought-provoking phenotypes and muscle-autonomous mechanisms of antisarcopenic action by exogenous glucocorticoid intermittence. Our findings challenge the current paradigm of glucocorticoids and muscle regulation, opening new myocyte-autonomous perspectives to counteract aging-related exercise intolerance and strength loss.

## Methods

### Sex as a biological variable.

We performed the bulk of our background-matched aged versus young mouse experiments using both males and females, analyzing and reporting the physiological, molecular, and histological assessments as sex disaggregated. We performed our unbiased omics-based screen unveiling PGC1α and Lipin1 as targets on the basis of RNA and epigenetics trends that were applicable to both sexes. Therefore, we used only males in subsequent KO-based proofs of requirement to minimize variability and overall mouse number used.

### Animal handling and treatments.

Euthanasia was performed through carbon dioxide inhalation followed by cervical dislocation and heart removal. Mice were maintained on a 14-hour light/10-hour dark cycle at a constant temperature of approximately 22°C and were given ad libitum access to chow and water. Aged (24 months old at the start of treatment) and young control (4 months old at the start of treatment) male and female mouse cohorts were obtained from the NIA Aged Rodent Colony and were on a C57BL/6JN background. Mice for mechanistic studies were obtained from The Jackson Laboratory and/or colleagues and were interbred. PGC1α-KO mice and WT PGC1α littermates were generated by crossing the 025750 and 009666 mouse lines, and Cre^–^ and Cre^+^ littermates were obtained from *Ppargc1a^WT/fl^* *HSA-MerCreMer^+/–^* matings. Analogously, we obtained Lipin1-KO mice and WT Lipin1 littermates from crossing *Lpin1^fl/fl^* (floxed exon7, complete protein loss; ref. 15) with the HSA-MCM mouse line. Both PGC1α- and Lipin1-KO mice were on the C57BL/6J background. Gene ablation was induced in mice starting at 3 months of age using i.p. injection (20 mg/kg per day for 5 days; MilliporeSigma, T5648) and then chow-mediated intake (40 mg/kg; Harlan, TD.130860) of tamoxifen for 14 days, followed by 14 days of washout (26). Prednisone treatment consisted of a once-weekly i.p. injection of 1 mg/kg prednisone (P6254, MilliporeSigma) (30). The injectable solution was diluted from a 5 mg/mL stock in DMSO (D2650; MilliporeSigma) in a 50 mL volume. Injections were done at the beginning of the light phase (ZT0; lights on). Pre- and post-noninvasive physiological assessments were conducted 72–24 hours before the first drug injection and 24 hours after the last injection. For epigenetic analyses, mice were sacrificed, and tissues were harvested 4 hours (ZT4) after the last prednisone injection in chronic or single-pulse treatments. For nonepigenetic experiments, tissues were harvested 24 hours after the last prednisone injection in chronic or single-pulse treatments, i.e., ZT0. All in vivo, ex vivo, and postmortem analyses were conducted by researchers blinded to the treatment groups.

### Analyses of muscle function, lean and muscle mass, and myofiber typing.

Our routine procedures concerning body composition, muscle function, muscle mass, and myofiber typing can be found as point-by-point protocols in ref. 48. All analyses were conducted by researchers blinded to the treatment.

### Respirometry with isolated mitochondria and muscle tissue.

Basal tissue OCR values were obtained from basal rates of oxygen consumption in muscle biopsies using the Seahorse XF HS Mini Extracellular Flux Analyzer platform (Agilent Technologies) under previously detailed conditions (30). The nutrients were as follows: 5 mM glucose and 1 mM palmitate-BSA (G7021, P0500, MilliporeSigma); and the inhibitors used were as follows: 0.5 mM rotenone plus 0.5 mM antimycin A (Agilent Technologies). The RCR values were obtained from mitochondria isolated from quadriceps muscle tissues using previously published procedures (27). Each well contained 2.5 μg mitochondria, with 20 μL of 50 mM ADP (MilliporeSigma, 01905), 50 μM oligomycin (MilliporeSigma,495455-10MG), 100 μM carbonyl cyanide-*p*-trifluoromethoxyphenylhydrazone (FCCP) (MilliporeSigma, C2920), and 5 μM rotenone (MilliporeSigma, 557368-1GM) and antimycin A (MilliporeSigma, A674-50MG) to yield final concentrations of 5,000, 50, 10, and 0.5 μM. The following nutrients were used: 0.5 mM pyruvate and 0.1 mM palmitoylcarnitine (P2256, 61251, MilliporeSigma). Seahorse measurements were conducted by researchers blinded to the treatment groups.

### Mitochondrial density, NMR, and mass spectrometry profiling.

The mtDNA/nDNA assay was performed on genomic DNA isolated using the Gbiosciences Omniprep kit (G-Biosciences, 786-136), using previously reported primers (49). For the MitoTracker assay, MitoTracker Green FM powder (Invitrogen, Thermo Fisher Scientific, M7514) was resuspended in 373 μL DMSO (Thermo Fisher Scientific, catalog BP231-100) to obtain a concentration of 200 μM. One microliter of this resuspension was added to 1 mL Mammalian Ringer’s Solution (Electron Microscopy Sciences, 11763-10) containing isolated myofibers from the FDB muscle of the mouse foot. The solution containing myofibers and MitoTracker was then pipetted into a 96-well plate (Corning, 9017) in increments of 200 μL. This plate was then read on the plate reader for fluorescence, with excitation set to 490 nm and emission to 516 nm. Values were then normalized to the protein content and assayed in each well after the MitoTracker assay through homogenization and a Bradford assay. NMR profiling was performed at the NMR Metabolomics Facility at CCHMC on quadriceps muscle tissues that were snap-frozen within 2 minutes after sacrifice, using previously reported protocols (50). Mass spectrometry (MS) profiling of hydrophilic metabolites through untargeted MS and with ^13^C isotope tracing ex vivo was performed at the Metabolomics Mass-Spec Core of Northwestern University (Chicago, Illinois, USA) using the conditions we detailed previously (30). For ^13^C-glucose tracing, we used 25 mU/mL insulin (Thermo Fisher Scientific, RP-10908) and 10 mM U-^13^C_6_-glucose (MilliporeSigma, 310808). NMR and MS analyses were performed by researchers blinded to the treatment groups.

### Matrix-assisted laser desorption/ionization MS.

Frozen quadriceps muscles were cryosectioned at 12 μm thickness using a Leica CM1860 cryostat. Cryosections were mounted onto indium tin oxide–coated glass slides (Delta Technologies) and then coated with 2′,4′,6′-trihydroxyacetophenone monohydrate at 10 mg/mL in 50:50 cyclohexane/methanol using a HTX TM Sprayer. Twenty passes were performed over each tissue at a spray volume of 50 mL/min and a nozzle temperature of 50°C. Once sprayed, the samples were individually wrapped in plastic bags and immediately transferred to –80°C. Matrix-associated laser desorption/ionization MS (MALDI MS) imaging was performed using a Q-Exactive HF mass spectrometer (Thermo Fisher Scientific) fitted with a MALDI/ESI Injector (Spectroglyph). MALDI-2 was used to enhance the analytical sensitivity for triglycerides and cholesteryl esters. Images were acquired at 20 μm voxel size using a pulse energy of approximately 6 mJ and a repetition rate of 30 Hz. The following Q Exactive HF MS Scan parameters were optimized for polar metabolites: polarity, negative; scan range, 350–1,500 *m/z*; resolution, 120,000; automatic gain control, off; maximum injection duration, 250 ms. ImageInsight (Spectroglyph) software was used for initial data visualization and to convert data files into imzML format for visualization and further processing in SciLS software (Bruker). All metabolite images produced were normalized to the total ion chromatogram.

### Metabolic cages and metabolic treadmill.

VO_2_ in baseline conditions (mL/h; expressed as aggregate values of L/day) was assessed via indirect calorimetry using the Prometheon Automated Phenotyping System (Sable Systems International) at the shared Metabolic Cage facility of the CCHMC Vet Services. Data collection started 24 hours after the last prednisone or vehicle injection and lasted for 5 days. Results are expressed as average values (all mice per group, all values per mouse, average of 5 days) over a circadian period, as well as in an ANCOVA (test for differences in regression lines, performed through CalR) (51), with average values of the active phase plotted against body mass values per mouse, as recommended in a prior publication (52). For VO_2_ analysis during aerobic exercise, an Oxymax Metabolic Treadmill (Columbus Instruments) was used, applying the stepwise speed increase protocol described previously to separate young versus aged mice on the basis of the slope of the VO_2_/workload curve and VO_2_ rates at baseline and submaximal and maximal workloads (40). The treadmill belt was angled at 10° uphill to match our regular treadmill conditions and to calculate work based on weight. Speed ramp-up was 3–5–8–12–15–17–20–23–25 m/min, with a stepwise increase every 5 minutes. Mice were assessed on the metabolic treadmill 24 hours after last vehicle or prednisone injection. The metabolic cage and metabolic treadmill assessments were performed by researchers blinded to the regimen or genotype.

### ChIP-Seq and RNA-Seq.

Muscle ChIP was performed using the conditions we previously reported (26). The primary antibody rabbit polyclonal anti-GR (Abclonal, A2164) was used. For ChIP, 100 μL Dynabeads M-280 (sheep anti-rabbit 11203D; Thermo Fisher Scientific) per sample was used. RNA-Seq was conducted on RNA extracted from quadriceps muscle. Total RNA was extracted from cryopulverized quadriceps muscles with TRIzol (Thermo Fisher Scientific, 15596026) and repurified using the RNeasy Mini Kit (QIAGEN, 74104). Both ChIP-Seq and RNA-Seq were performed at the CCHMC DNA Sequencing Core, generating 20 million or more high-quality, 100-base-length read pairs per sample. Details regarding library preparation and sequencing are available in our GEO data sets (GSE245227, GSE245493). ChIP-Seq analysis was conducted using standard commands in HOMER software, version 4.10 (53), after aligning fastq files to the mm10 mouse genome using bowtie2 (54). RNA-Seq analysis was performed using kallisto, version 0.43.1 (55). PCA was conducted using ClustVis (56). Heatmaps of peak density were imaged with TreeView3 (57). Peak tracks were imaged through the WashU epigenome browser. Gene ontology pathway enrichment was conducted using the Gene Ontology analysis tool (58).

### WB, qPCR, OPP, and hydroxyproline assays.

Protein analysis was performed on approximately 50 mg total lysates from whole quadriceps muscles homogenized in general protein buffer, i.e., PBS supplemented with 1 mM CaCl_2_, 1 mM MgCl_2_ (MilliporeSigma, C1016, M8266), and protease and phosphatase inhibitors (Roche, 04693232001, 04906837001). For blocking and stripping solutions, StartingBlock and RestorePLUS buffers (Thermo Fisher Scientific, 37543, 46430) were used. The following primary antibodies (all diluted 1:1,000 for overnight incubation at 4°C) were used: rabbit anti-PGC1α (ABClonal, A12348), rabbit anti-GR (ABClonal, A2164), mouse anti-puromycin (Developmental Studies Hybridoma Bank [DSHB], PMY-2A4), rabbit anti-GLUD1 (ABClonal, A7631), rabbit anti-GLUL (ABClonal, A21856), rabbit anti-GOT2 (ABClonal, A19245), rabbit anti-GPT2 (ABClonal, A23670), rabbit anti–Lipin1 (ABClonal, A14111), rabbit anti-GAPDH (ABClonal, AC027), rabbit anti-LC3B (ABClonal, A19665), rabbit anti–pink 1 (ABClonal, A11435), rabbit anti-Mfn2 (ABClonal, A19678), rabbit anti-Fis1 (ABClonal, A19666), and total OXPHOS Rodent WB Antibody Cocktail (Abcam, ab110413). HRP-conjugated donkey anti-rabbit or anti-mouse (Santa Cruz Biotechnology, sc-2313, sc-2314) secondary antibodies were used (diluted 1:5,000 for 1 hour incubation at room temperature). Counterstaining for loading the control was performed using Ponceau (MilliporeSigma, P7170) and/or GAPDH staining. Blots were developed with SuperSignal Pico (Thermo Fisher Scientific, 34579) using the iBrightCL1000 developer system (Thermo Fisher Scientific, A32749) with automatic exposure settings. Western blot gels and membranes were run and transferred in parallel and/or stripped for multiple antibody–based staining for densitometric analyses. Protein density was analyzed using the Gel Analysis tool in ImageJ software (NIH) (59) and expressed as fold changes versus control samples.

*O*-propargyl-puromycin (OPP) fluorometry was performed, adapting the regular instructions for the Click-iT Plus OPP Alexa Fluor 488 Protein Synthesis Assay Kit (Thermo Fisher Scientific, C10456) to live myofibers isolated from FDB muscle using previously reported conditions (60). Protein puromycinylation was assessed in gastrocnemius muscle tissue through anti-puromycin Western blotting of whole protein lysates 30 minutes after i.p. puromycin injection (0.040 μmol/body weight in grams; MilliporeSigma, P8833).

For reverse transcription quantitative PCR (RT-qPCR) assays, total RNA was reverse transcribed using 1× qScript Supermix (95048, QuantaBio), and qPCRs were conducted in triplicate using 1× Sybr Green Fast qPCR mix (ABclonal, RK21200) and 100 nM primers on a CFX96 qPCR machine (Bio-Rad; thermal profile: 95°C for 15 seconds, 60°C for 30 seconds, 40 times, melting curve). Primers were selected among validated primer sets from the following Massachusetts General Hospital (MGH) PrimerBank IDs: GPT2-27805389a1; GLUD1-6680027a1; GLUL-31982332a1; GOT2-6754036a1; LIPIN1-27923941a1); and from the published PGC1a1 and PGc1a4 primmer sets (7) and mitochondrial DNA quantification primers (49).

Hydroxyproline content was measured in frozen gastrocnemius muscles as previously described (29). Results are reported as micromoles of hydroxyproline per 100 milligrams tissue. The negative control was an age-matched gastrocnemius sample from an uninjected WT mouse; the positive control was an age-matched gastrocnemius sample from an uninjected X chromosome–linked muscular dystrophy (mdx) mouse (The Jackson Laboratory, JAX 013141).

### Luciferase assays in C2C12 myoblasts.

Luciferase plasmids containing regulatory fragments were obtained by subcloning genomic sequences into the pGL4.23 backbone (Promega, E8411), conserving the genomic orientation of the target sequences with regard to transcriptional orientation. Regions of 250 bp encompassing the canonical GRE (ACAnnnTGT) were selected. Control sequences (containing the GRE) and GRE-deleted sequences (missing only the GRE) were generated through custom gBlocks from Integrated DNA Technologies (IDT). Plasmids were then transfected into C2C12 (American Type Culture Collection [ATCC], CRL-1772) via Lipofectamine 3000 (Thermo Fisher Scientific, L3000001), together with a *Renilla* luciferase as the internal normalizer. Luciferase signal was then measured as *Renilla*-normalized Fluc luminescence at 48 hours after either 25 μg/mL prednisone or vehicle using the Dual-Glo assay (Promega, E2920). The tested regions (mm39 coordinates as chromosome, strand, start, end) were as follows: within the GR peak on the *Pparcg1a* distal TSS (chr5 + 51712172 51712421); within the GR peak on the *Pparcg1a* proximal TSS (chr5 + 51723374 51723623); within the GR peak on the *Lpin1* promoter (chr12 + 16668459 16668708).

### Muscle lipidomics.

Internal standards (Splash Lipidomix, Avanti Polar Lipids) were added to tissue homogenates, and lipids were extracted as described previously (61). Lipid identification and quantitation were performed using previously reported methods (62–64). Lipid species with coefficients of variation of greater than 20% between the technical replicates were excluded from further analysis. Analysis for heatmaps was based on peak area values normalized to milligrams of tissue.

### AAV preparation and injection.

Approximately 70%–80% confluent HEK293T cells (AAVpro 293T Cell Line and 632273 AAVpro 293T Cell Line, both from Takara) in DMEM (Cytiva Life Sciences, SH30022.01) supplemented with 2% bovine growth serum (BGS) (Cytiva Life Sciences) and 1.0 mM sodium pyruvate were triple-transfected with pHelper (Cell Biolabs, 340202), pAAV-GOI [Vector Builder (VB230317-1361ncv; pAAV[Exp]-CMV>mPpargc1a[NM_008904.3]*-V5:WPRE); (VB230317-1364xmj; pAAV[Exp]-CMV>mPpargc1a_isoform4-Myc:WPRE)], and pAAV Rep-Cap (1A-Myo; recloned from a previously published sequence [ref. 38], gift from the Molkentin laboratory (Cincinnati Children’s Hospital, Cincinnati, Ohio, USA) plasmids using PEI, Linear, MW 250,000 (PolySciences) in 40 T 150 mm cell culture plates. Eighteen hours after transfection, the medium was changed to DMEM supplemented with 1% BGS, 1.0 mM sodium pyruvate, and 1× MEM Nonessential Amino Acid Solution (MilliporeSigma, M7148). Approximately 96 hours after transfection, the media and cells were collected and processed separately. Cells were lysed using repeated freeze/thaw cycles a minimum of 5 times in 1× Gradient Buffer (0.1 M Tris, 0.5 M NaCl, 0.1 M MgCl_2_). The cell debris was then treated with benzonase endonuclease at 0.65 μL/5 mL (100,000 units, MilliporeSigma, 1037731010) for at least 1 hour. The homogenates were cleared from the debris by centrifugation. AAVs were precipitated from the cell medium with PEG 8000. The PEG-precipitated AAV was collected by centrifugation, and the AAV pellet was resuspended in 1× gradient buffer. Media and cell AAVs were combined and loaded onto an iodixanol (OptiPrep Density Gradient Medium, MilliporeSigma, D1556250) gradient at 15%, 25%, 40% and 60% in 1× gradient buffer and subjected to ultracentrifugation. The 40% iodixanol layer, containing the AAV particles, was extracted, and a buffer exchange into 2× PBS/10 mM MgCl_2_ was performed using Centrifugal Filters (30,000 nominal molecular weight limit [NMWL], 4.0 mL sample volume; MilliporeSigma, UFC803024, and 100,000 NMWL, 15.0 mL sample volume; MilliporeSigma, UFC910024). Primers binding within the CMV region of AAV-GOI ITR (forward: GTTCCGCGTTACATAACTTACGG; reverse: CTGCCAAGTGGGCAGTTTACC) were used to measure the virus titer with qPCR. Before releasing the viral DNA from the particles, all extraviral DNA was removed by digestion with DNase I. Then, the viral DNA was released by proteinase K digestion. For injection, 10^12^ vg was diluted in 50 μL saline and injected r.o. into anesthetized mice (isoflurane 1.5%) the same day of the prednisone injection at week 10 of the treatment.

### Statistics.

Statistical analyses were performed using GraphPad Prism, version 9.2.0 (GraphPad Software). The Pearson-D’Agostino normality test was used to assess data distribution. When comparing data groups for 3 related variables (age, drug, treatment time; genotype, drug, treatment time), 3-way ANOVA was used with pre-/post-sample matching and Šidák’s multiple comparisons. When comparing data groups for 2 related variables, 2-way ANOVA was used with Šidák’s multiple comparisons (treatment vs. age effect; treatment vs. KO effect). A *P* value of less than 0.05 was considered statistically significant. When data points were less than 10, the data were presented as single values (dot plots, histograms). Tukey’s distribution bars or violin plots were used to emphasize the data range distribution for more than 10 data points per pool. The SEM is shown for curves. Values for each plotted point are reported as upper and lower lines.

### Study approval.

Mice were housed in a pathogen-free facility in accordance with the American Veterinary Medical Association (AVMA) and under protocols fully approved by the IACUC of Cincinnati Children’s Hospital Medical Center (approval nos. 2022-0020 and 2023-0002). Consistent with the ethics approvals, all efforts were made to minimize suffering of the animals.

### Data availability.

The RNA-Seq and ChIP-Seq data sets reported here are available in the NCBI’s Gene Expression Omnibtu (GEO) database (GEO GSE245227 and GSE245493). Data values for all graphs can be found in the Supporting Data Values file.

## Author contributions

ADP, K McFarland, K Miz, HBD, KP, FEAS, HL, CW, HJC, NSB, AJM, and BP conducted experiments and acquired and analyzed data. DPM and, BNF provided reagents and revised the manuscript. MQ provided reagents, designed research, analyzed data, and wrote the manuscript.

## Supplementary Material

Supplemental data

Unedited blot and gel images

Supporting data values

## Version 1 05/03/2024

In-Press Preview

## Version 2 06/03/2024

Electronic publication
