# Supplementary material for: Intermittent glucocorticoid treatment improves muscle metabolism via the PGC1α/Lipin1 axis in an aging-related sarcopenia model
Source: J Clin Invest. 2024 May 3;134(11):e177427. doi: 10.1172/JCI177427 (PMC11142738; doi:10.1172/JCI177427)
Supplement: Supplemental data [file jci-134-177427-s086.pdf]

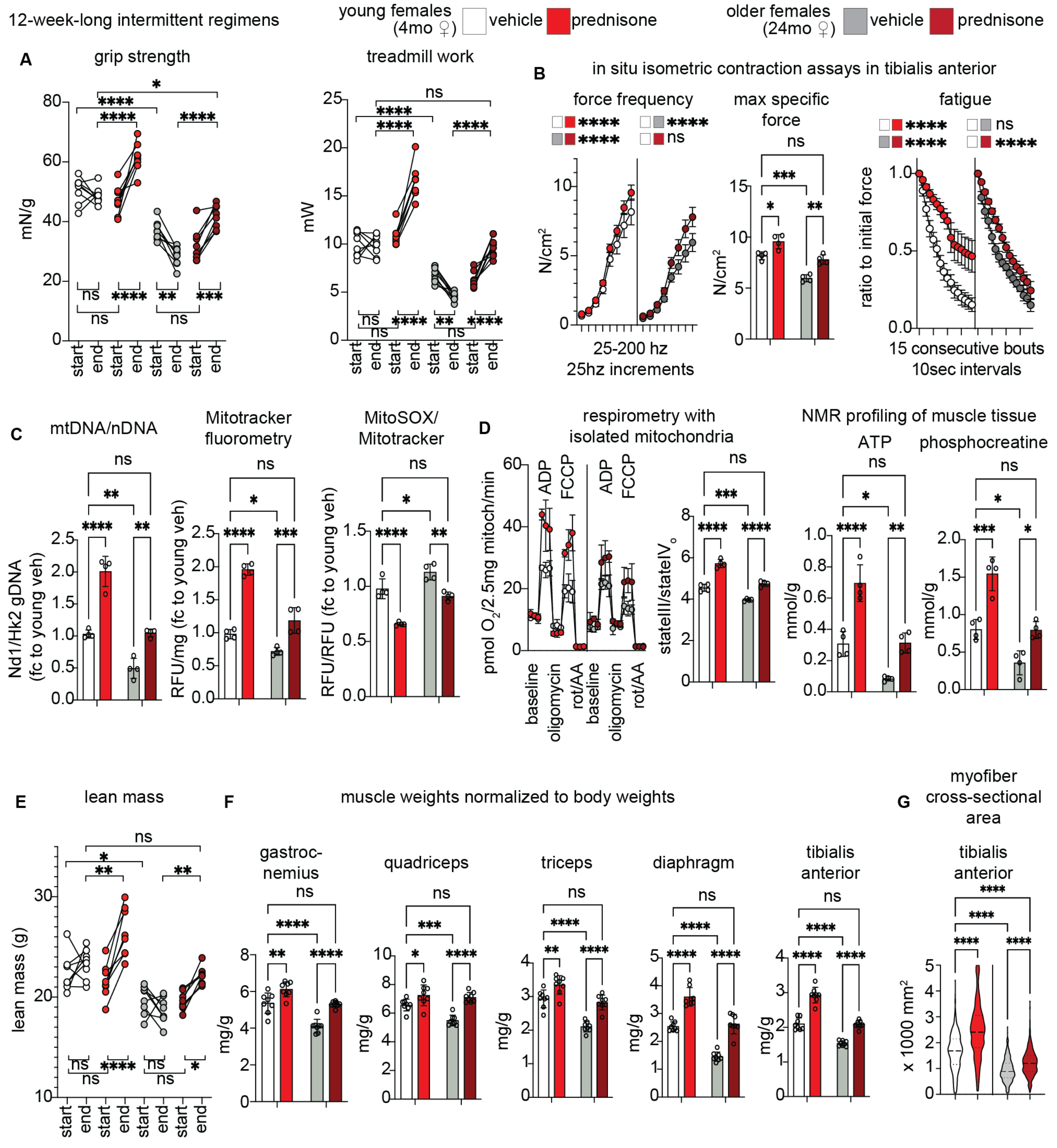

Supplementary Figure 1. Related to Figure 1. Additional analyses in young/older female mice. Parallel analyses in age- and background-matched WT females showed analogous treatment effects on overall function and muscle energy-mass, as compared to the trends in male mice shown in Figure 1. N=4-8/group; histograms and curves report mean±s.e.m., pre-post plots report each subject trend, violin plots indicate mean and 25-75 percentiles; (start-end) pre/post-paired 3w ANOVA + Sidak; (endpoint) 2w ANOVA + Sidak: *, P<0.05; **, P<0.01; ***, P<0.001; ****, P<0.0001.

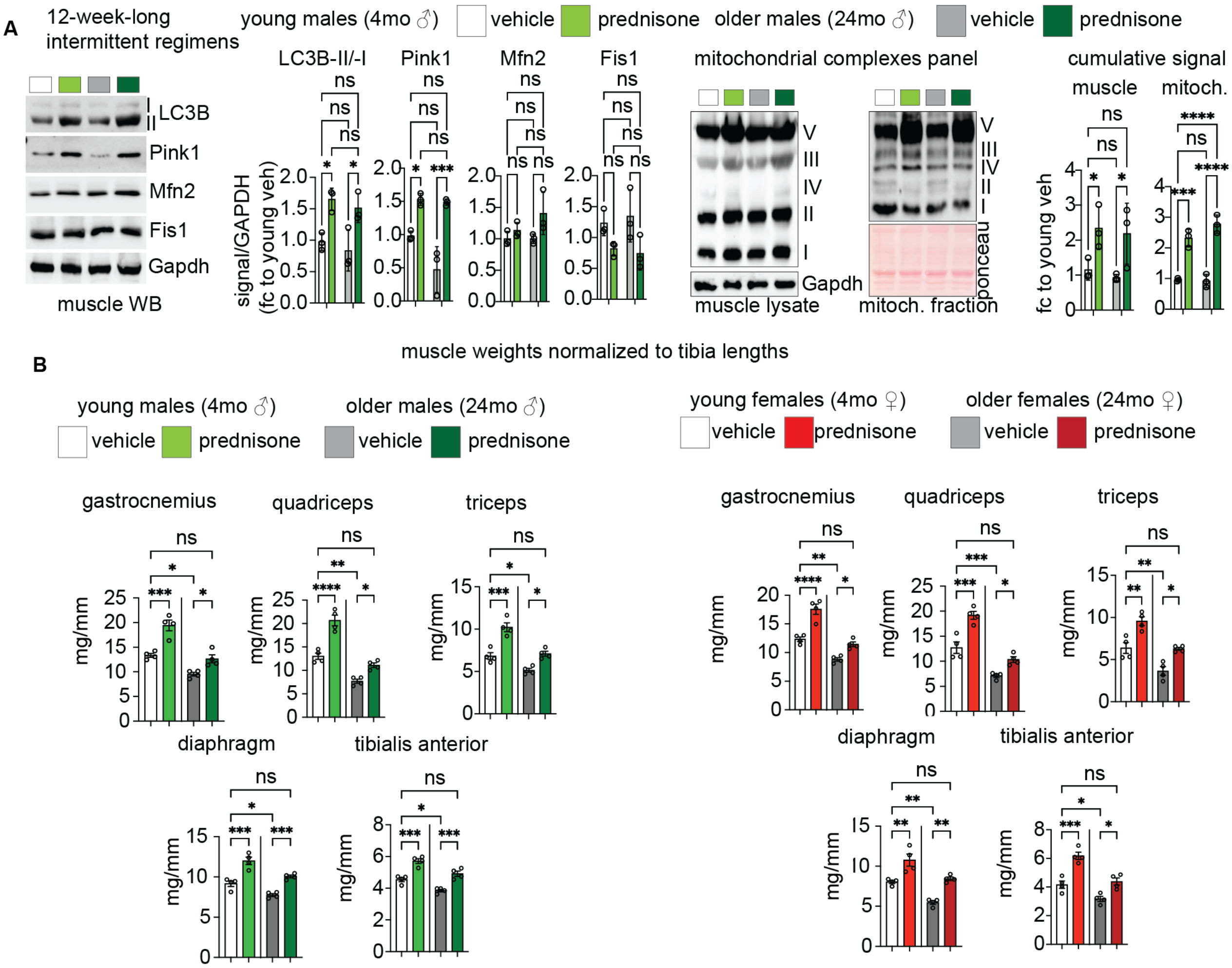

Supplementary Figure 2. Related to Figure 1. Protein markers of autophagy, mitophagy, mitochondrial fusion and fission, and muscle weight/tibia length values. (A) Treatment increased LC3B-II/-I and Pink1 protein levels in young and aged muscles, while slight changes in Mfn2 and Fis1 were not significant. Cumulative mitochondrial complex signal was increased by treatment in quadriceps muscle, in either whole lysates or mitochondrial fractions. **(B)** Parallel analyses in the mouse subsets where we recorded tibia lengths showed analogous aging and treatment effects on muscle weight/tibia length values, as compared to the trends shown by muscle weight/body weight analyses. N=4/group; histograms report mean±s.e.m.; 2w ANOVA + Sidak: *, P<0.05; **, P<0.01; ***, P<0.001; ****, P<0.0001.

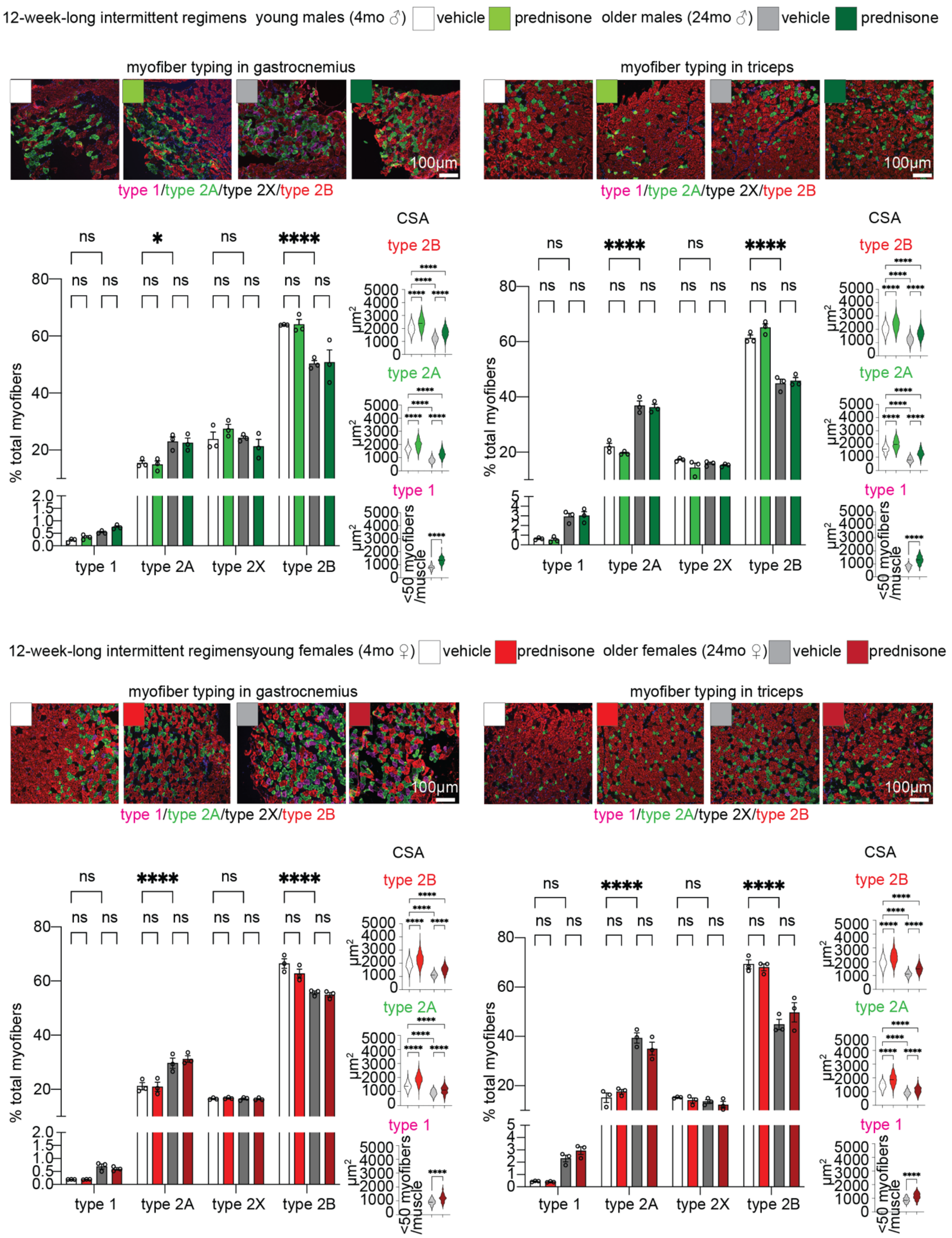

Supplementary Figure 3. Related to Figure 1. Treatment did not change myofiber type distribution. No significant effects of treatment were observed on top of the age-related shifts in both male and female mice in two locomotory muscles, gastrocnemius (hindlimbs) and triceps (forelimbs). Aging decreased CSA in type 2B and 2A myofibers, and treatment increased CSA in all stained myofiber types (3mice/group for all; 2B: 400/muscle; 2A: 200/muscle; 1: 50/muscle). N=3/group; histograms report mean±s.e.m., violin plots indicate mean and 25-75 percentiles;2w ANOVA + Sidak, (type 1) Welch's t-test: *, $P<0.05$; **, $P<0.01$; ***, $P<0.001$; ****, $P<0.0001$.

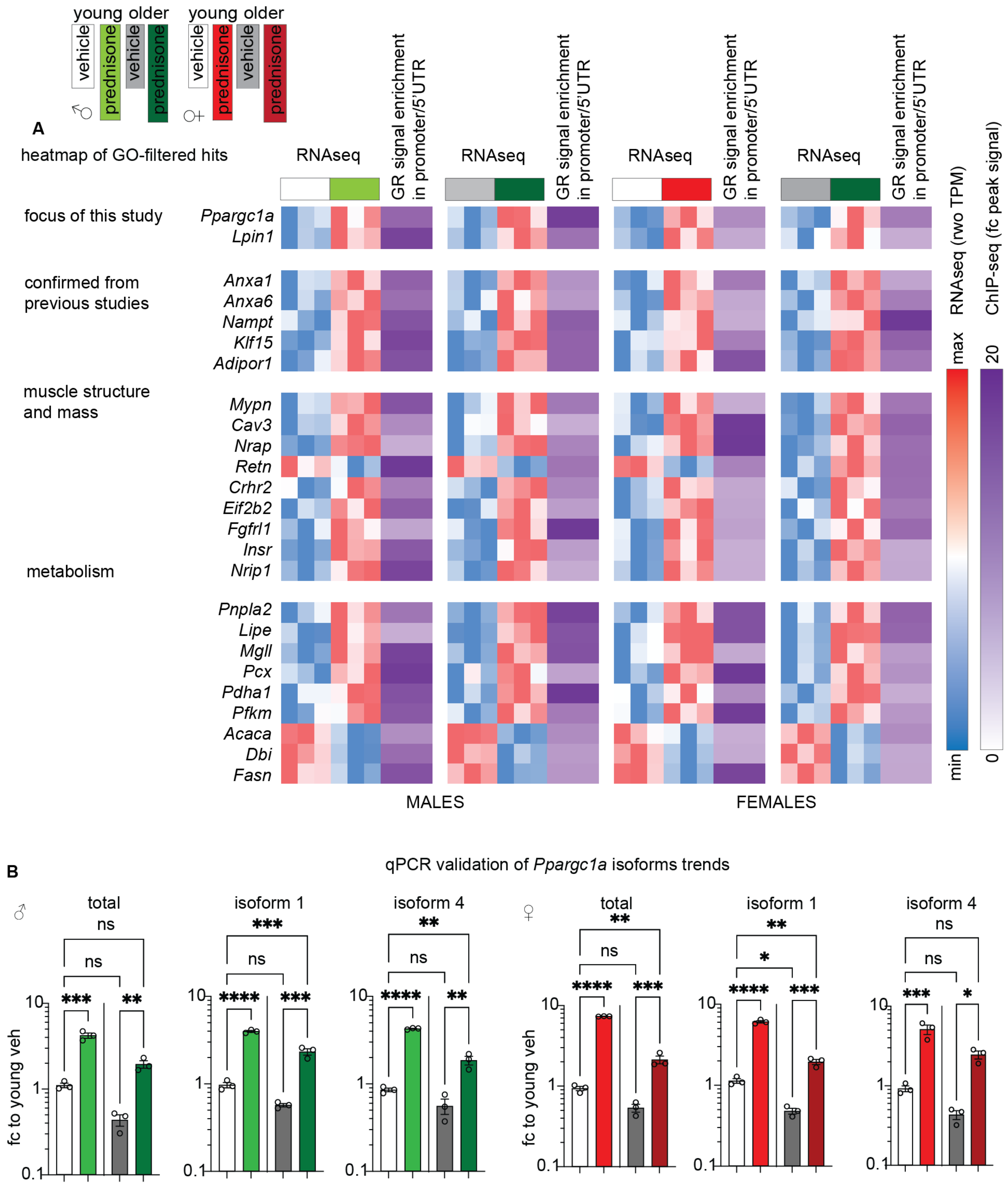

Supplementary Figure 4. Related to Figure 2. Additional analyses regarding ChIP-seq X RNA-seq overlay and Ppargc1a hits. (A) Heatmap summarizing RNA and GR signal trends for all GO-filtered hits according to sex/age groups. **(B)** qPCR analysis in quadriceps muscle RNA samples confirmed the RNA-seq trends in total Ppargc1a and isoforms 1/4 expression. N=3/group; histograms report mean±s.e.m.; 2w ANOVA + Sidak, (type 1) Welch's t-test: *, P<0.05; **, P<0.01; ***, P<0.001; ****, P<0.0001.

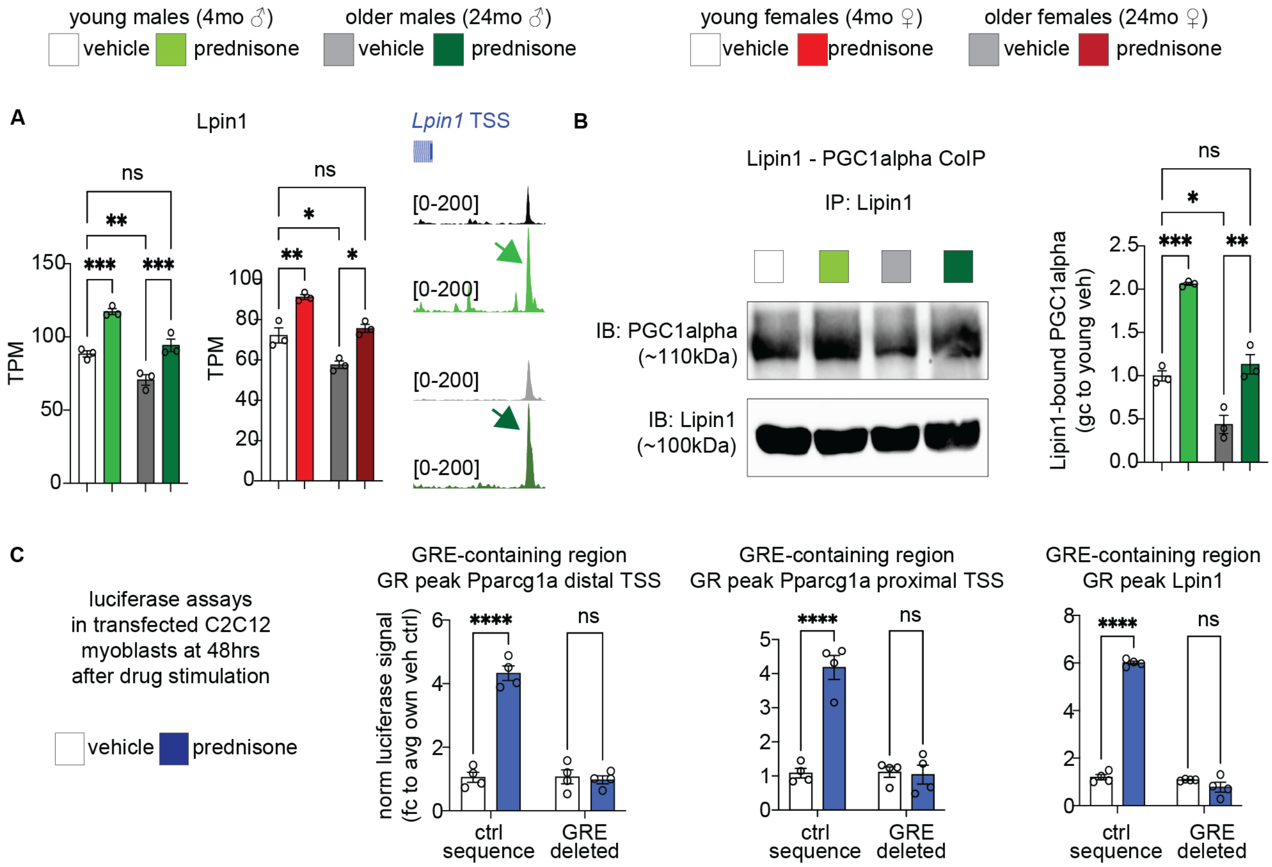

Supplementary Figure 5. Related to Figure 2. Additional analyses regarding Ppargc1a-Lpin1 hits. (A) *Lpin1* expression was upregulated by treatment and rescued to young-like levels in older muscles in both males and females. Treatment increased the GR peak on *Lpin1* promoter in both age groups. **(B)** CoIP analysis from control and treated quadriceps muscles showed increased Lipin1-PGC1alpha interaction after treatment counteracting the aging-related effect. **(C)** Luciferase assays with the GRE-containing vs GRE-deleted regions identified by ChIP-seq on proximal and distal *Pparcg1a* promoters and on the *Lipin1* promoter. N=3 mice/group (a-b), 4 transfection replicates/group in (c); histograms report mean±s.e.m.; 2w ANOVA + Sidak: *, P<0.05; **, P<0.01; ***, P<0.001; ****, P<0.0001.

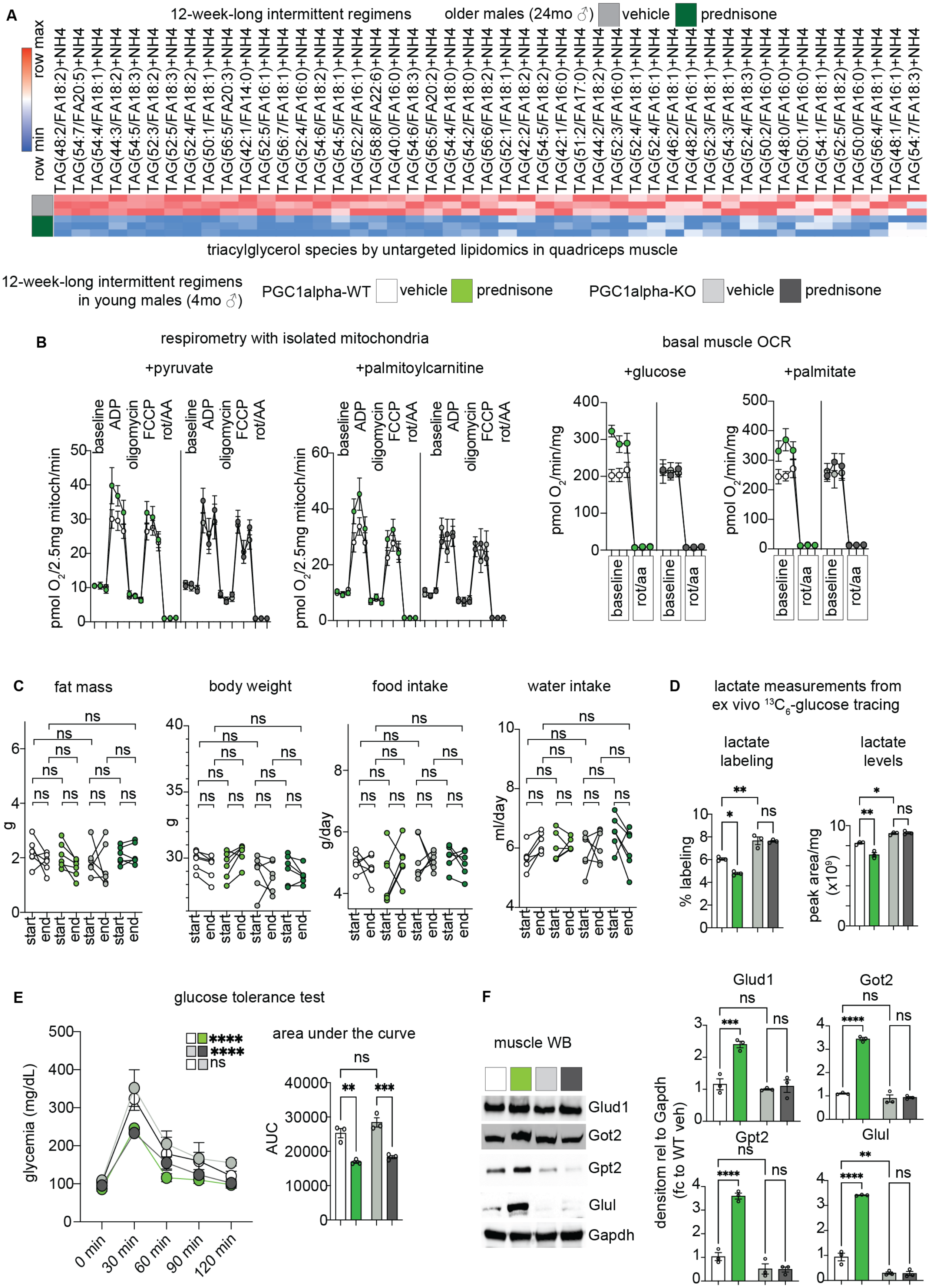

Supplementary Figure 6. Related to Figures 3-4. Additional analyses in aged mice and with inducible PGC1alpha ablation in myofibers. (A) Untargeted lipidomics revealed a pervasive downregulation in muscle triacylglycerol content. (**B**) Respirometry curves for mitochondria and muscle biopsies, with calculated RCR and

basal OCR values in Figure 3D. (**C**) No changes in fat mass, body weight, food intake and water intake were significant by muscle PGC1alpha ablation or treatment, at baseline or at endpoint (food and water intake were monitored for one week at each time point). **(D)** In PGC1alpha-WT muscle, treatment decreased carbon labeling and overall levels of lactate, while both parameters were increased in both treated and untreated PGC1alpha-KO muscle. **(E)** Treatment increased glucose tolerance regardless of myofiber PGC1alpha presence. **(F)** WB confirming the PGC1alpha-dependent upregulation of Glud1, Got2, Gpt2, Glul levels in muscle downstream of treatment. N=3-6/group; histograms and curves report mean±s.e.m., pre-post plots report each subject trend, violin plots indicate mean and 25-75 percentiles; (start-end) pre/post-paired 3w ANOVA + Sidak; (endpoint) 2w ANOVA + Sidak: *, $P<0.05$; **, $P<0.01$; ***, $P<0.001$; ****, $P<0.0001$.

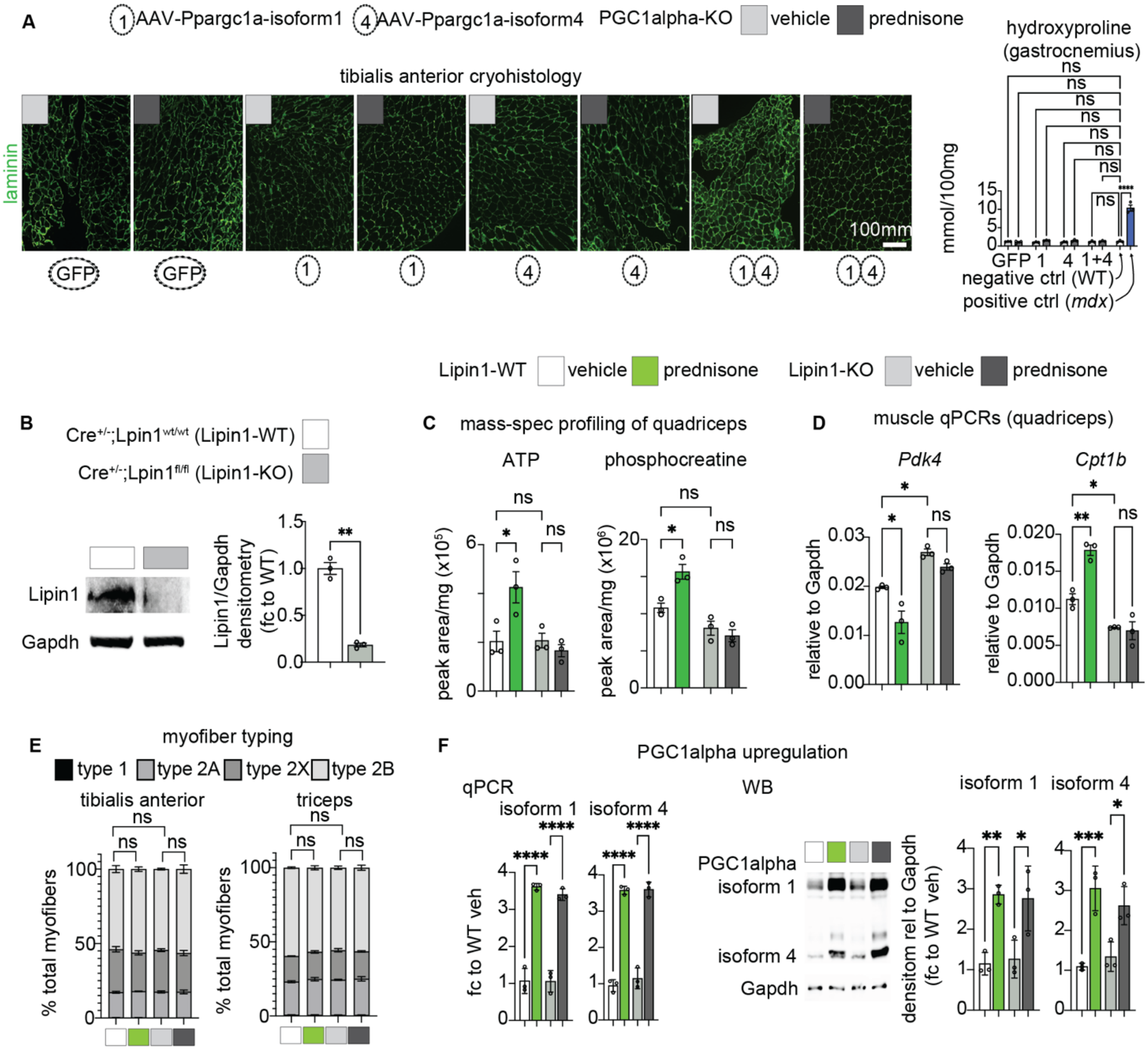

Supplementary Figure 7. Related to Figure 5. Additional analyses with MyoAAV-PGC1alpha isoforms and inducible Lipin1 ablation in myofibers. (A) Laminin staining of tibialis anterior cryosections did not reveal obvious muscle architecture impairments, nor did hydroxyproline dosing in gastrocnemius revealed any increases in fibrosis deposition in PGC1alpha-KO mice injected with MyoAAVs, using WT and mdx dystrophic muscles as negative and positive control respectably. **(B)** Lipin1-KO validation. **(C)** Lipin1 was required for the treatment effect on muscle ATP and phosphocreatine levels. **(D)** In line with the treatment-driven increases in respirometry with pyruvate and palmitoylcarnitine (Figure 5E), we found that in WT muscle treatment decreased *Pdk4* and increased *Cpt1b* expression compared to vehicle. Consistently with decreased respirometry in KO vs WT muscles (Figure 5F), we found that, compared to WT control, KO muscles showed upregulated *Pdk4* and downregulated *Cpt1b* with negligible treatment effects. **(E)** No sizeable changes in myofiber typing by genotype or treatment. **(F)** Treatment increased expression of both isoforms 1 and 4 of PGC1alpha in muscle independently from myofiber Lipin1 presence. N=3/group; histograms report mean±s.e.m.; Welch's t-test (b), 2w ANOVA + Sidak (a,c): *, P<0.05; **, P<0.01; ***, P<0.001; ****, P<0.0001.
