# Supplementary material for: Intermittent glucocorticoid treatment improves muscle metabolism via the PGC1α/Lipin1 axis in an aging-related sarcopenia model
Source: J Clin Invest. 2024 May 3;134(11):e177427. doi: 10.1172/JCI177427 (PMC11142738; doi:10.1172/JCI177427)

Original blots

Suppl Fig 5B

IP: Lipin1

IB: Lipin1 IB: PGC1alpha

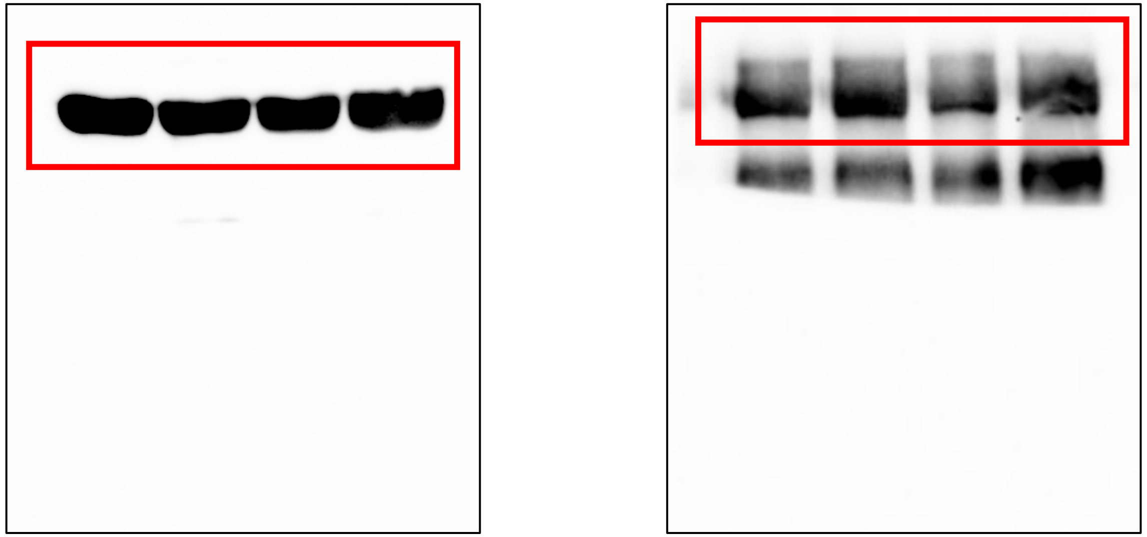

Figure 3F

puromycin

gapdh

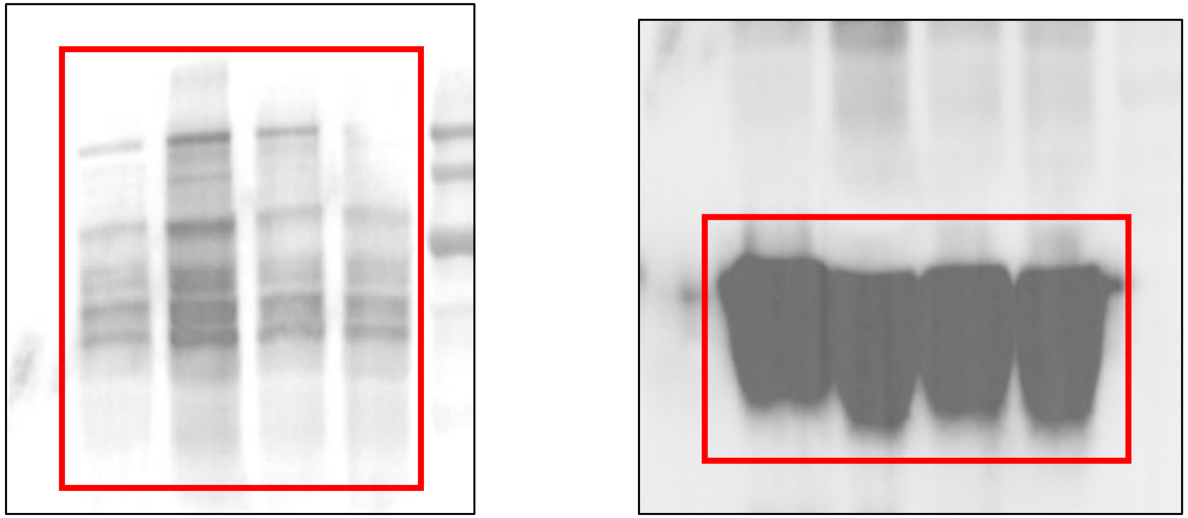

Suppl Fig 7B

Lipin1 Gapdh

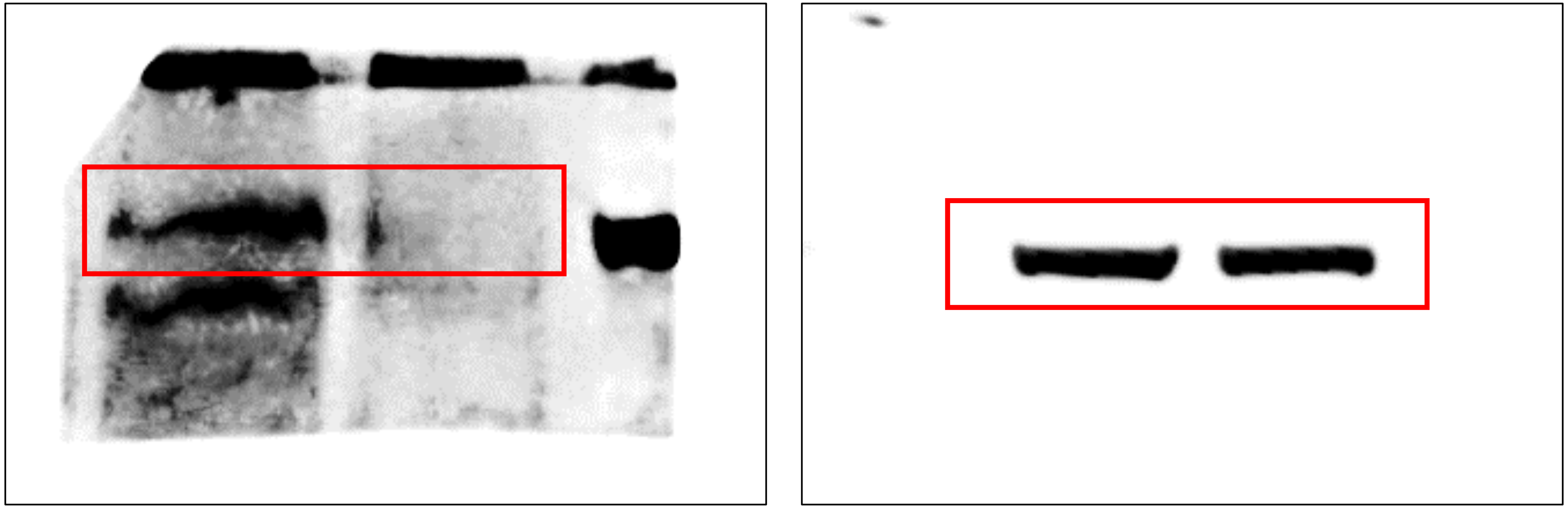

Suppl Fig 7F

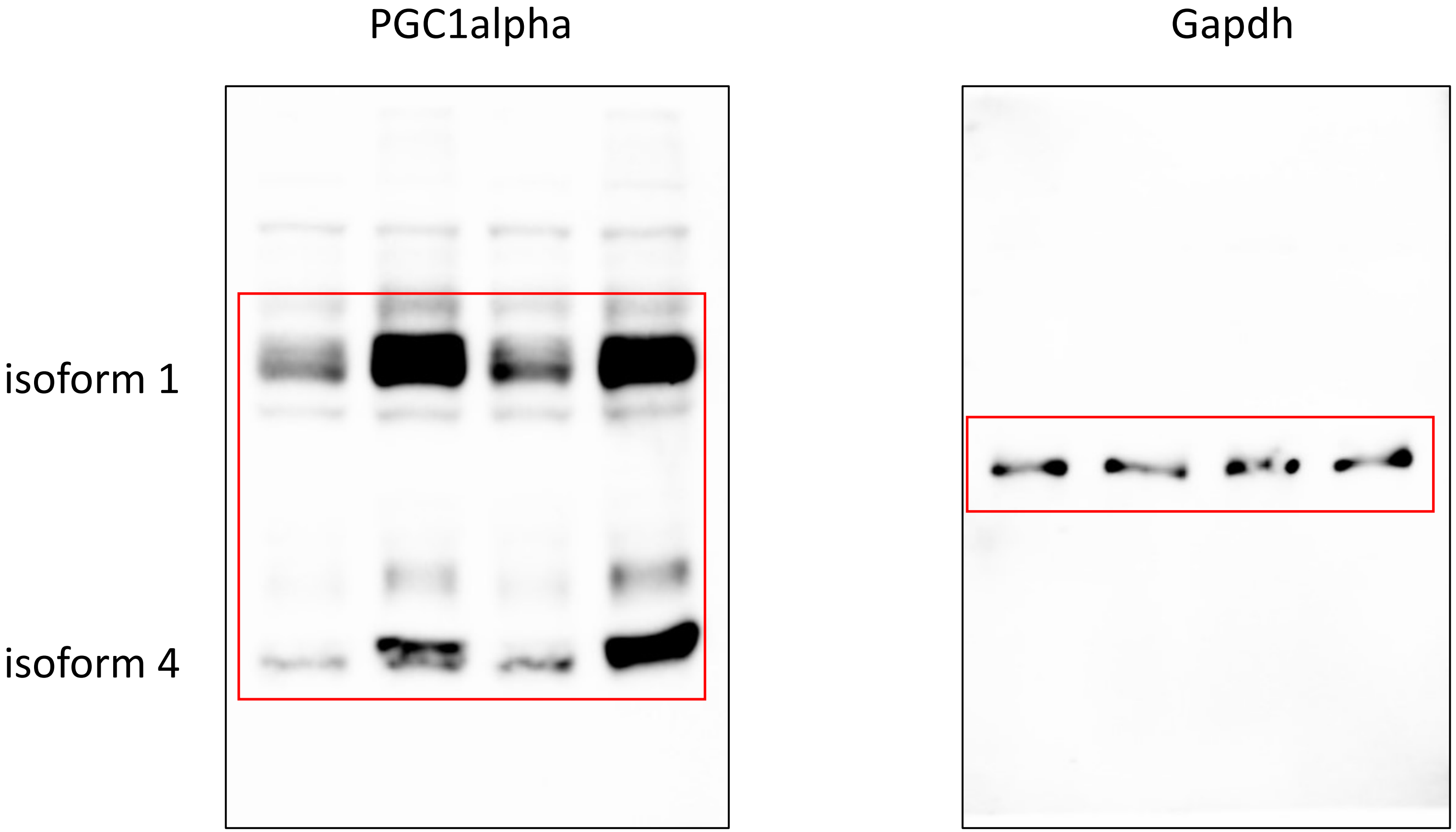

Suppl Fig 6F

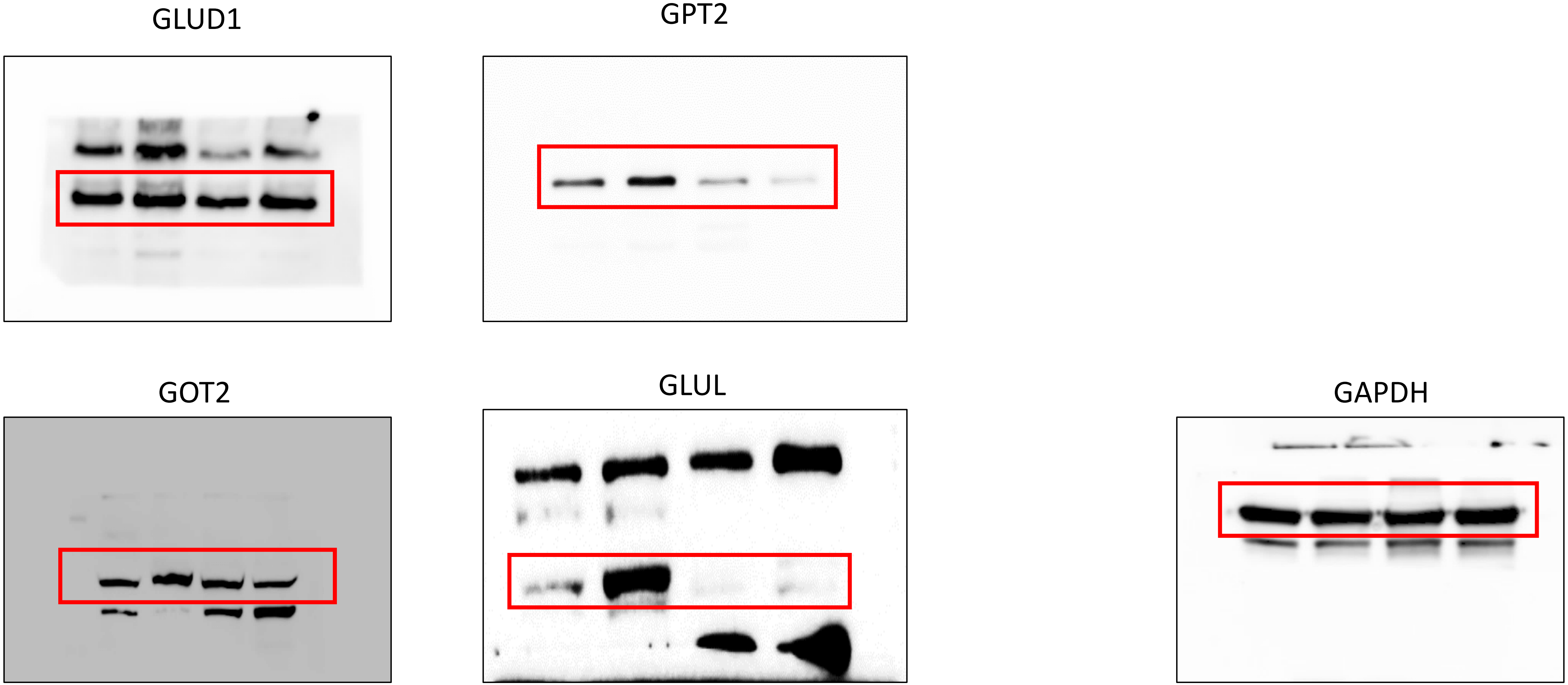

Suppl Fig 2A - left

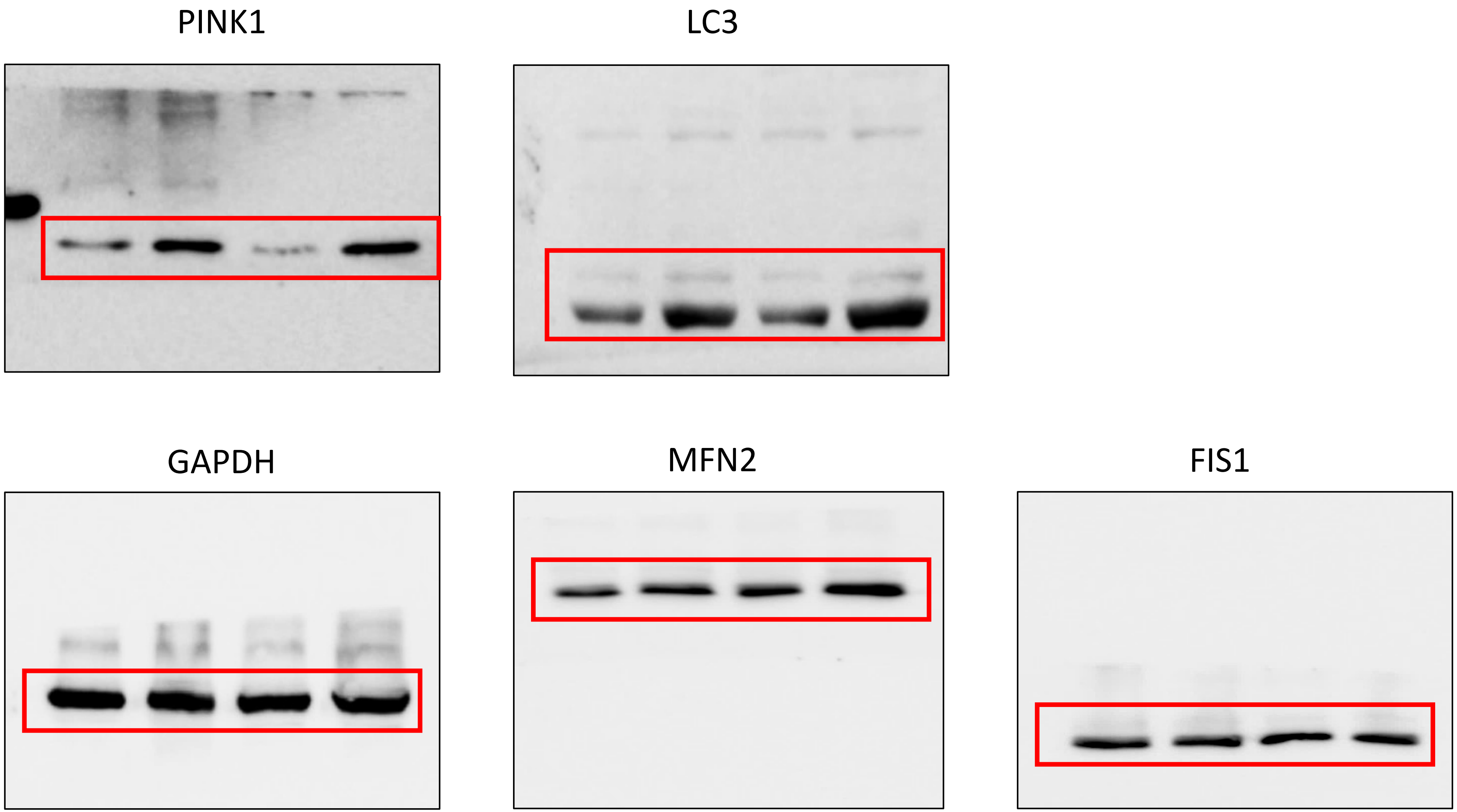

Suppl Fig 2A - right

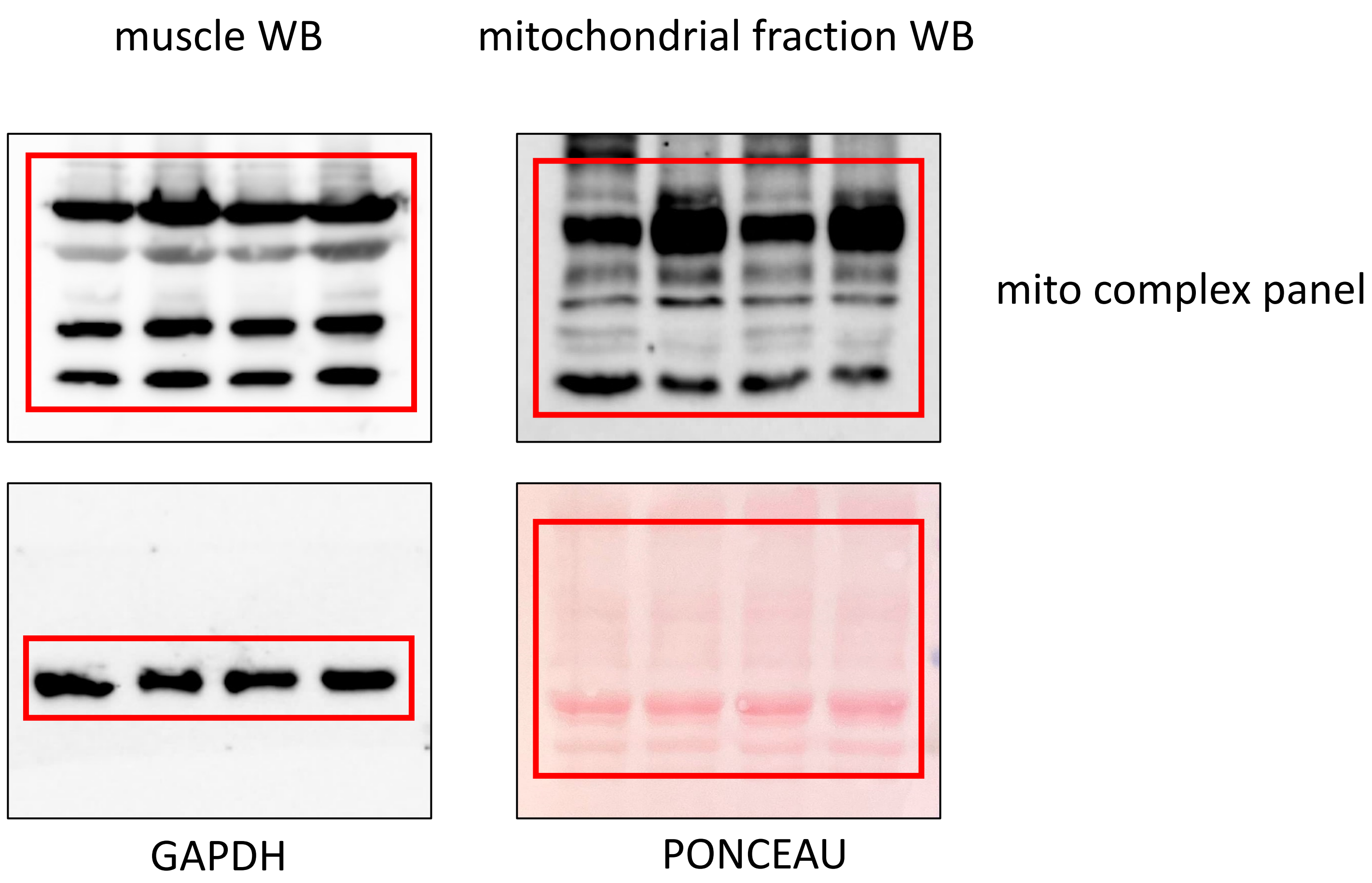

Supplement: Unedited blot and gel images [file jci-134-177427-s087.pdf]
